# Integrating High-Resolution Coastal Acidification Monitoring Data Across Seven United States Estuaries

**DOI:** 10.3389/fmars.2021.679913

**Published:** 2021-08-19

**Authors:** Nicholas A. Rosenau, Holly Galavotti, Kimberly K. Yates, Curtis C. Bohlen, Christopher W. Hunt, Matthew Liebman, Cheryl A. Brown, Stephen R. Pacella, John L. Largier, Karina J. Nielsen, Xinping Hu, Melissa R. McCutcheon, James M. Vasslides, Matthew Poach, Tom Ford, Karina Johnston, Alex Steele

**Affiliations:** 1 Ocean and Coastal Management Branch, Office of Wetlands Oceans and Watersheds, United States Environmental Protection Agency, Washington, DC, United States,; 2 United States Geological Survey, St. Petersburg Coastal and Marine Science Center, St. Petersburg, FL, United States,; 3 Casco Bay Estuary Partnership, Cutler Institute, University of Southern Maine, Portland, ME, United States,; 4 Ocean Process Analysis Laboratory, Institute for the Study of Earth, Oceans, and Space, University of New Hampshire, Durham, NH, United States,; 5 United States Environmental Protection Agency Region 1, Boston, MA, United States,; 6 Pacific Coastal Ecology Branch, Pacific Ecological Systems Division, Office of Research and Development, United States Environmental Protection Agency, Newport, OR, United States,; 7 Coastal and Marine Sciences Institute, University of California, Davis, Bodega Bay, CA, United States,; 8 Estuary & Ocean Science Center, San Francisco State University, Tiburon, CA, United States,; 9 Harte Research Institute for Gulf of Mexico Studies, Texas A&M University-Corpus Christi, Corpus Christi, TX, United States,; 10 Barnegat Bay Partnership, Ocean County College, Toms River, NJ, United States,; 11 NOAA Northeast Fisheries Science Center, Milford, CT, United States,; 12 The Bay Foundation, Los Angeles, CA, United States,; 13 Ocean Monitoring and Research Group, Los Angeles County Sanitation District (LACSD), Whittier, CA, United States

**Keywords:** coastal acidification, ocean acidfication, estuary, autonomous sensor, carbon dioxide, pH, dissolved oxygen, National Estuary Program

## Abstract

Beginning in 2015, the United States Environmental Protection Agency’s (EPA’s) National Estuary Program (NEP) started a collaboration with partners in seven estuaries along the East Coast (Barnegat Bay; Casco Bay), West Coast (Santa Monica Bay; San Francisco Bay; Tillamook Bay), and the Gulf of Mexico (GOM) Coast (Tampa Bay; Mission-Aransas Estuary) of the United States to expand the use of autonomous monitoring of partial pressure of carbon dioxide (*p*CO_2_) and pH. Analysis of high-frequency (hourly to sub-hourly) coastal acidification data including *p*CO_2_, pH, temperature, salinity, and dissolved oxygen (DO) indicate that the sensors effectively captured key parameter measurements under challenging environmental conditions, allowing for an initial characterization of daily to seasonal trends in carbonate chemistry across a range of estuarine settings. Multi-year monitoring showed that across all water bodies temperature and *p*CO_2_ covaried, suggesting that *p*CO_2_ variability was governed, in part, by seasonal temperature changes with average *p*CO_2_ being lower in cooler, winter months and higher in warmer, summer months. Furthermore, the timing of seasonal shifts towards increasing (or decreasing) *p*CO_2_ varied by location and appears to be related to regional climate conditions. Specifically, *p*CO_2_ increases began earlier in the year in warmer water, lower latitude water bodies in the GOM (Tampa Bay; Mission-Aransas Estuary) as compared with cooler water, higher latitude water bodies in the northeast (Barnegat Bay; Casco Bay), and upwelling-influenced West Coast water bodies (Tillamook Bay; Santa Monica Bay; San Francisco Bay). Results suggest that both thermal and non-thermal influences are important drivers of *p*CO_2_ in Tampa Bay oxygen, National Estuary Program and Mission-Aransas Estuary. Conversely, non-thermal processes, most notably the biogeochemical structure of coastal upwelling, appear to be largely responsible for the observed *p*CO_2_ values in West Coast water bodies. The co-occurrence of high salinity, high *p*CO_2_, low DO, and low temperature water in Santa Monica Bay and San Francisco Bay characterize the coastal upwelling paradigm that is also evident in Tillamook Bay when upwelling dominates freshwater runoff and local processes. These data demonstrate that high-quality carbonate chemistry observations can be recorded from estuarine environments using autonomous sensors originally designed for open-ocean settings.

## INTRODUCTION

An increase in the anthropogenic concentration of carbon dioxide (CO_2_) dissolved in marine waters is putting stress on marine systems. This process, known as ocean acidification, refers to the changes in carbonate chemistry to the ocean that result from the absorption of increasing atmospheric CO_2_, primarily from human fossil fuel combustion (Doney et al., 2009). The chemical effects of CO_2_ absorption (e.g., reduced pH) are naturally buffered by the ocean’s carbonate system via reaction with carbonate ions (CO_3_^2−^) and bicarbonate ions (HCO_3_^−^). With the continued uptake of CO_2_ by the oceans, this buffering results in an increase in the partial pressure of CO_2_ in water (*p*CO_2_) and a decrease in pH, dissolved CO_3_^2−^ and HCO_3_^−^ concentrations, and associated carbonate mineral saturation states.

In coastal and estuarine environments, understanding carbonate chemistry is further complicated by the interaction and dynamism of multiple co-occurring chemical, biological, and physical processes, relative to the open ocean, operating at various spatial and temporal scales (Hofmann et al., 2011; Waldbusser and Salisbury, 2014). Coastal and estuarine systems are susceptible to local and regional acidification due to eutrophication, air-water flux of CO_2_, coastal upwelling, changes in freshwater inflow, stratification, and other factors. Changes to the carbonate chemistry of marine waters can adversely affect the ability of shellfish and other calcifying organisms to build or maintain their calcium carbonate (CaCO_3_) shells and skeletons (Talmage and Gobler, 2010; Gazeau et al., 2013; Waldbusser et al., 2015; Feely et al., 2016). Changes in *p*CO_2_ and pH have also been shown to impact other physiological processes, including species growth, survival, fertilization, embryonic/larval development, and behavior (Fabry et al., 2008; Pörtner, 2008; Doney et al., 2009; Kroeker et al., 2013; Gledhill et al., 2015). Impairments to fish and shellfish physiology can lead to adverse impacts to the ecology of marine and estuarine systems. The effects of ocean and coastal acidification are already being seen in fish and shellfish aquaculture across the country (e.g., Barton et al., 2015; Mabardy et al., 2015), potentially threatening an important industry in many coastal communities (Barton et al., 2012; Ekstrom et al., 2015; Clements and Chopin, 2017).

**FIGURE 1 |:**
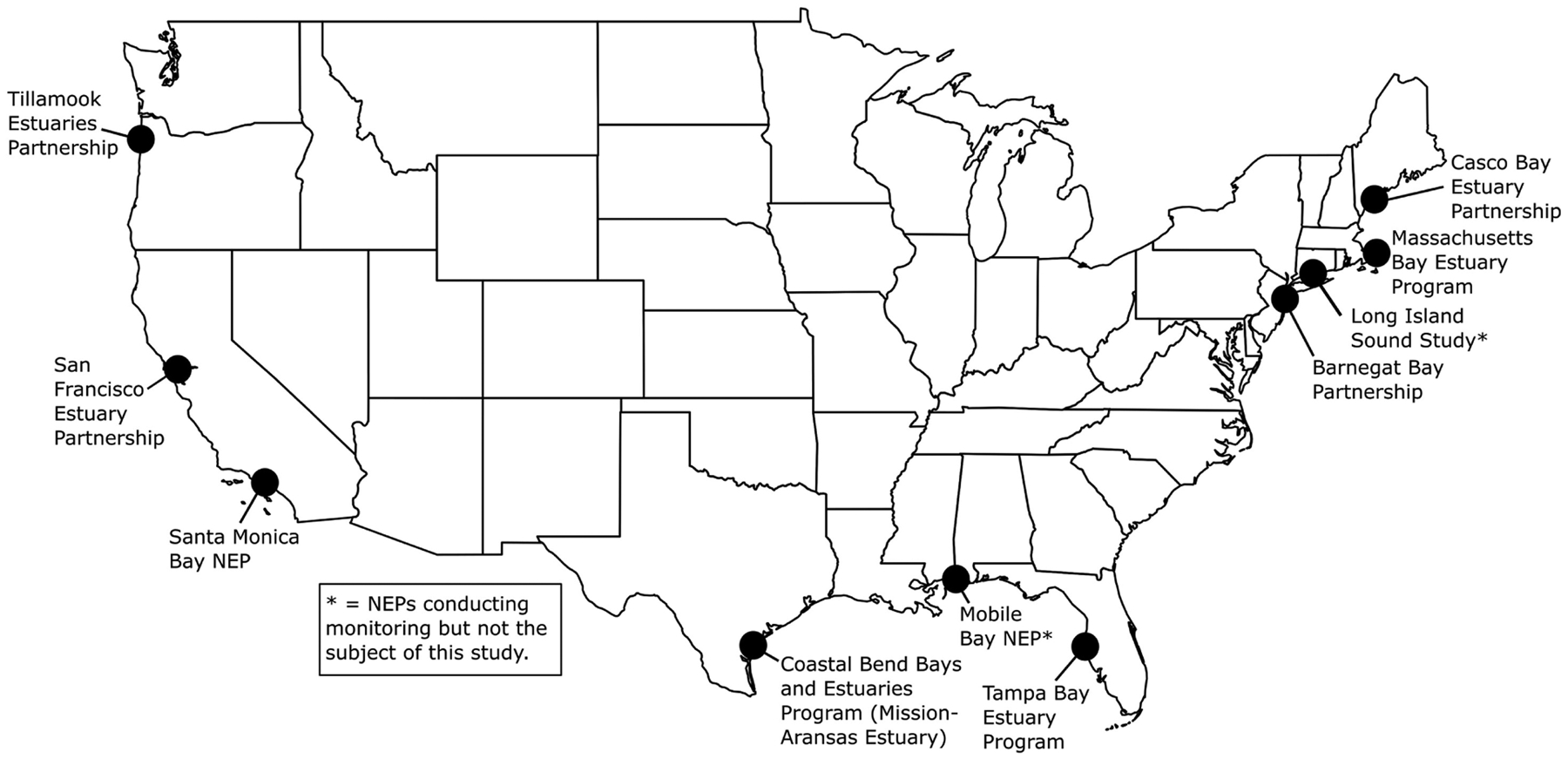
United States Environmental Protection Agency’s (EPA’s) National Estuary Programs (NEPs) with coastal acidification monitoring data included in this study. See Figure 2 for more details on deployments. Mobile Bay NEP, Long Island Sound Study and Massachusetts Bay NEP (denoted with asterisks) are conducting monitoring but are not the subject of this study. Map created by NR.

High-resolution, multiparameter monitoring via autonomous sensors is important for characterizing the carbonate chemistry of estuarine waters and distinguishing the drivers of coastal acidification. These monitoring data can shed light on the vulnerability of these systems to acidification and guide specific mitigation and adaptation strategies such as using seagrass to decrease *p*CO_2_, improving aquaculture techniques to buffer hatchery systems, and adapting nutrient management plans (Kelly et al., 2011; Strong et al., 2014; Chan et al., 2016). Autonomous sensors have been used extensively in the open ocean to monitor ocean acidification; however, their deployment in coastal and estuarine waters is challenging due to rapid variation over large ranges in temperature, salinity and chemical composition, biofouling, sensor drift, and other factors (Sastri et al., 2019). The United States Environmental Protection Agency’s (EPA’s) National Estuary Program (NEP)^1^ has expanded the use of autonomous carbonate chemistry sensors in estuarine environments. The NEP is a place-based program to protect and restore the water quality and ecological integrity of estuaries of national significance. The 28 estuaries that make up the NEP are located along the Atlantic, Gulf, and Pacific coasts, and in Puerto Rico. The programs are in a variety of institutional settings, including federal, state, and local agencies, universities, and individual nonprofit organizations. Beginning in 2015, EPA funded nine NEPs to purchase autonomous *p*CO_2_ and pH sensors to characterize carbonate chemistry conditions and form a more mechanistic understanding of coastal acidification in their estuaries (Figure 1). EPA’s Office of Research and Development (ORD) Pacific Ecological Systems Division conducted the monitoring in Tillamook Bay. Ten NEPs and their partners have been collecting hourly and sub-hourly *in situ p*CO_2_ and pH data and have worked to optimize monitoring methods and data analysis procedures (EPA, 2021). Data from seven of these NEPs are discussed here.

The objective of this study is to describe and compare coastal acidification data from seven coastal systems around the United States in terms of typical values (e.g., median, range), seasonality and co-variability to begin to understand natural variability in carbonate chemistry parameters within and across a range of environmental settings. Continued monitoring and collection of long-term, high-resolution data are needed to detect real trends in coastal acidification, distinguish these from background variability, and determine the relative influence of the drivers and impact of acidification in these different systems.

## NEP COASTAL ACIDIFICATION MONITORING PROGRAM

### Water Body Characteristics

**FIGURE 2 |:**
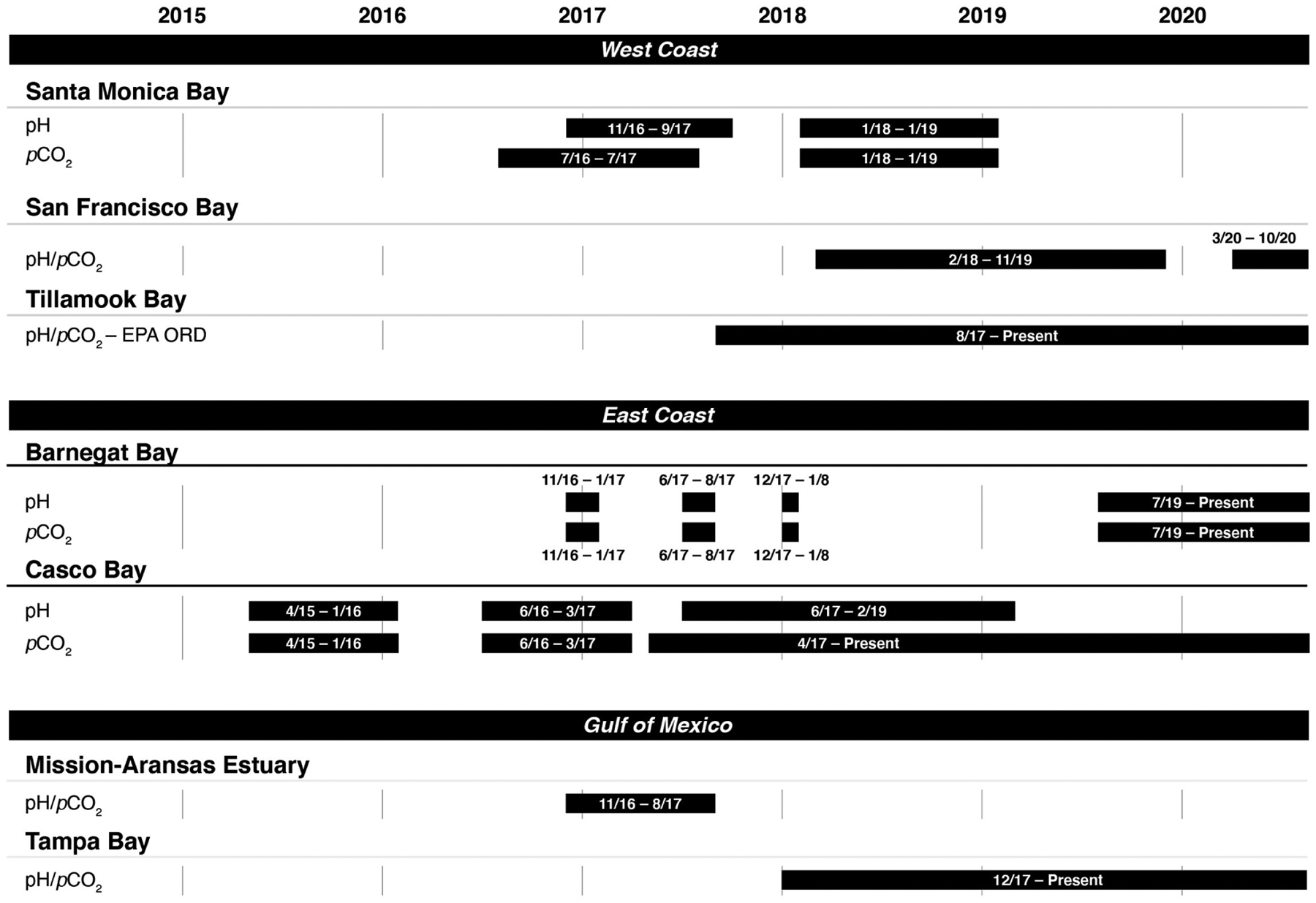
Instrument deployment timelines for measuring pH and the partial pressure of carbon dioxide (*p*CO_2_) in seven of the United States Environmental Protection Agency’s (EPA’s) National Estuary Program sites.

The seven NEPs and their partners conducting coastal acidification monitoring that are discussed in this report include two NEPs on the East Coast (Casco Bay Estuary Partnership, ME, United States; Barnegat Bay Partnership, NJ, United States), two NEPs in the Gulf of Mexico [GOM (Tampa Bay Estuary Program, FL, United States; Coastal Bend Bays and Estuaries Program/Mission-Aransas Estuary, TX, United States)] and three NEPs on the West Coast (San Francisco Bay Estuary Partnership, CA, United States; Santa Monica Bay NEP, CA, United States; Tillamook Estuaries Partnership, OR, United States; Figures 1, 2). The NEPs of Long Island Sound (Long Island Sound Study), Massachusetts Bay (Massachusetts Bay NEP) and Mobile Bay (Mobile Bay NEP) also conduct coastal acidification monitoring with autonomous *p*CO_2_ and pH sensors; however, data are unavailable, and therefore, are not the subject of this study. In Massachusetts Bay and Mobile Bay, sensors had yet to be deployed prior to beginning this analysis so data was unavailable for inclusion. In Long Island Sound, researchers were already conducting an in-depth analysis of their monitoring data prior to initiation of this report and wanted to complete their analysis before making the data available for inclusion in a multi-estuary synthesis. The water bodies studied vary in geographic location, size, environmental stressors, coastal dynamics and processes, and local economic interests (Supplementary Table 1). Watershed size ranges from 1,428 km^2^ (Tillamook Bay) to 5,698 km^2^ (Tampa Bay). Population size ranges from ~7,500 individuals in the Tillamook Bay watershed to ~7,000,000 individuals, in the San Francisco Bay watershed, and land use ranges from more urbanized (Santa Monica Bay, Tampa Bay) to more undeveloped and forested (Tillamook Bay). Santa Monica Bay has the greatest average water depth at 95 meters (m), while Tillamook Bay has an average depth of ~1.4 m. Tidal height ranges from 0.15 m in Barnegat Bay to ~3 m in Casco Bay.

The NEPs on the East Coast are characterized by cool waters with some coastal upwelling. Casco Bay is fed by twelve significant lake and river systems and also has a large tidal influence. The Bay is dotted with roughly 785 islands, islets, and exposed ledges. Casco Bay has an important shellfish restoration and aquaculture industry, including lobster and clam fisheries. The Barnegat Bay estuarine system is composed of three shallow, micro-tidal bays: Barnegat Bay, Manahawkin Bay, and Little Egg Harbor. A nearly continuous barrier island complex runs along the eastern edge of Barnegat Bay, separating it from the Atlantic Ocean. The estuary has an upwelling center off Little Egg Inlet, and it is also fed by low pH and alkalinity freshwater. It is located in an urban watershed and the Upper Barnegat Bay is highly eutrophic. There are a number of shellfish aquaculture and restoration projects throughout the watershed, in addition to the historic hard clam fishery.

The NEPs in the GOM region are in a transition zone between warm-temperate and tropical biogeographic provinces, and are characterized by warm, productive waters. Tampa Bay is a large, shallow, open-water estuary stretching 1,030 km^2^ at high tide. It is influenced by the mixing of GOM waters with freshwater flow from more than 100 tributaries, dozens of meandering, brackish-water creeks and four major rivers. On average, Tampa Bay is only ~4 m deep; however, manmade shipping channels have been dredged to allow large ships safe passage to the Port of Tampa and other bay harbors. It has economically important shellfish and finfish populations. Tampa Bay is specifically examining the role of seagrass in protecting marine species from the harmful effects of coastal acidification. The monitoring location in the Mission Aransas Estuary in the Port Aransas Ship Channel (i.e., Aransas Pass tidal inlet) connects the Gulf coastal water with the Aransas, Corpus Christi, and Redfish bays. As secondary bays to Corpus Christi and Aransas bays, Nueces and Copano bays receive freshwater input from the Nueces River and Mission/Aransas rivers, respectively. The high alkalinity of freshwater flows into Mission Aransas Estuary are an important characteristic of the bay (Hu et al., 2015).

The NEPs in the West Coast region are characterized as having cooler, deeper waters with prominent coastal upwelling (Leinweber and Gruber, 2013; Raimonet and Cloern, 2017). Santa Monica Bay is influenced by both freshwater inflows, primarily from the Ballona Creek, Malibu Creek, and Topanga Creek watersheds, and coastal upwelling. Santa Monica Bay is especially unique amongst these seven estuaries because it is more of an open, deep coastal site on a narrow continental shelf rather than an enclosed estuary. The Santa Monica Bay Foundation is specifically looking at the feasibility of restored kelp forests as a pH refuge for marine life from the harmful effects of ocean acidification. The San Francisco Bay monitoring sites are located within a tidal excursion of the mouth of the bay, at the interface between Central Bay (outer embayment) and San Pablo Bay (North Bay). There is a high range of salinity at this location in which at low tide, there is an estuarine water signal influenced by freshwater flows, while at high tide, there is an ocean water signal. There is no commercial shellfish production in San Francisco Bay due to historic water quality issues. The herring fishery is the only commercial fishery inside San Francisco Bay; however, there is an extensive nursery habitat for the economically important Dungeness crab fishery, restoration efforts for the native Olympic oyster, and concern for migrating salmonid and other endangered species in the upper estuary. The Tillamook Estuary is a relatively shallow estuary with maintained jetties and channels less than 6.7 m. It is located in a more rural watershed with high nutrient inputs from dairy and timber farms and wastewater treatment plants. The estuary is influenced by periods of high river discharge. Tillamook Estuary has commercial shellfish aquaculture (including oysters and bay clams) as well as extensive recreational fisheries. The monitoring site in Tillamook Estuary is located at the Port of Garibaldi, which is a commercial fish offloading dock near the mouth of the estuary as well as in the vicinity of a wastewater treatment outfall.

### Monitoring Timeline

Casco Bay was the first program to begin coastal acidification monitoring in 2015. The other NEPs began monitoring in 2016 or 2017. Figure 2 shows the time periods in which monitoring data were collected in each NEP as of publication of this study. Most continue to collect data as of June 2020.

### Deployments and Measurements

**FIGURE 3 |:**
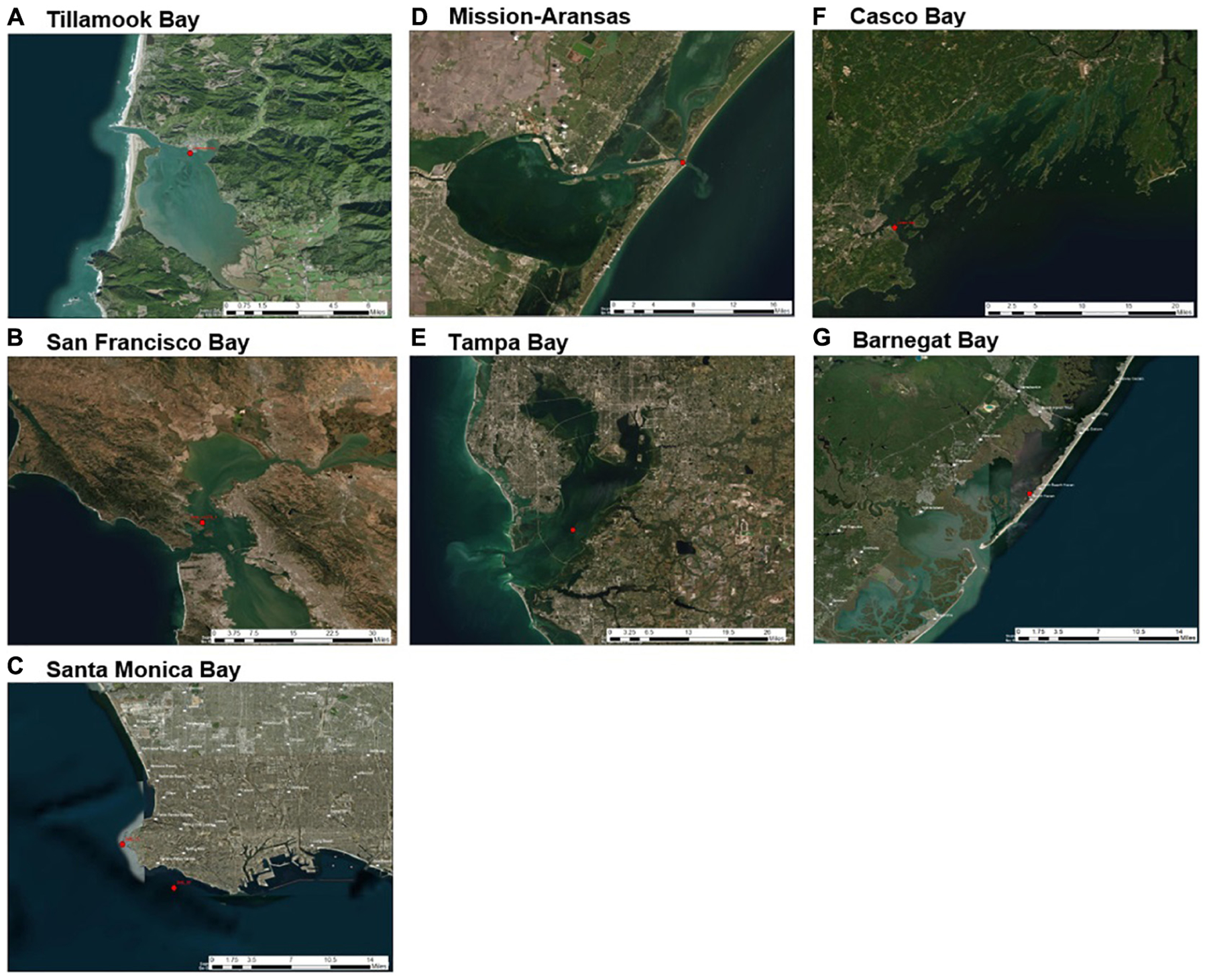
Sensor deployment locations (red dots) in each water body of United States Environmental Protection Agency’s (EPA’s) National Estuary Program (NEP) sites discussed in this study: **(A)** Tillamook Bay; **(B)** San Francisco Bay; **(C)** Santa Monica Bay; **(D)** Mission-Aransas Estuary; **(E)** Tampa Bay; **(F)** Casco Bay; **(G)** Barnegat Bay. Satellite imagery base layer accessed at https://services.arcgisonline.com/ArcGIS/rest/services/World_Imagery/MapServer. Map image is the intellectual property of ESRI and is used herein under license. Copyright© ESRI 2021 and its licensors. All rights reserved.

The monitoring approaches used by each program varied, including deployment methods, types of equipment, and discrete sampling methods, and were driven largely by existing capacities as well as regional influences and scientific interests. Coastal acidification monitoring equipment was deployed on fixed, land-based structures (e.g., docks, piers, and pilings), as well as water-based moorings (Supplementary Table 2, Figure 3). Differences in deployment locations were undoubtedly reflected in the recorded data. For example, shallower, nearshore sites were expected to preserve a stronger diel biological signal and would be more sensitive to freshwater runoff or land-based pollutants as compared to further offshore sites like deeper water Santa Monica Bay and San Francisco Bay deployments. Each of the seven NEPs collected *in situ* measurements of *p*CO_2_, pH, temperature, salinity, and DO (Tables 1, 2). However, DO data for Mission–Aransas Estuary were collected at a different depth in the water column and therefore were not evaluated.

### Instruments

Several different autonomous sensors were used in the estuaries. Specifications of the various instruments, including instrument resolution, precision, and operating ranges, are shown in Table 2. The sensors used for measuring pH include the Sea-Bird SeaFET and Sea-Bird SeapHOx. The SeaFET pH sensor is an ion-sensitive field effect transistor (ISFET), which is more precise and stable over time compared to pH sensors that use glass electrodes. The pH range for the SeaFET is 6.5 to 9.0. The SeapHOx integrates a SeaFET pH sensor with additional sensors that measure temperature, salinity, pressure, and DO. The SeapHOx also includes an internal water pump and anti-fouling technology. Both the SeaFET and SeapHOx have internal battery power and data logging capabilities. For measurement of *p*CO_2_, four of the NEPs used the Sunburst SAMI-CO_2_ and the remaining NEPs used Pro-Oceanus CO_2_-Pro or Moored Autonomous *p*CO_2_ (MAPCO_2_) systems. The Sunburst SAMI-CO_2_ uses a colorimetric method to measure the partial pressure of CO_2_ from 200 to 600 μatm typically, although extended range calibrations are available by request through the manufacturer. The Pro-Oceanus CO_2_-Pro and MAPCO_2_ measure *p*CO_2_ using an infrared CO_2_ detector. See Sutton et al. (2014) for additional details on the MAPCO_2_ system. Supporting data (e.g., temperature, salinity, pressure, DO), are measured using a variety of Sea-Bird, Yellow Springs Instrument (YSI), or Aanderaa instruments. All pH data reported by the NEPs are on the total pH scale, (pH_*T*_) allowing direct comparison.

## METHODS

### Data QA/QC and Validation

The protocols for assessing data quality and validating *in-situ* measurements varied across the NEPs. Because they are all federally funded programs, all NEPs incorporate comparably stringent Quality Assurance/Quality Control (QA/QC) procedures in their data screening and validation protocols as outlined in a Quality Assurance Project Plan (QAPP)^2^. This includes collecting data that comply with EPA rules for surface-water-quality monitoring programs and water-quality assessments to support decisions related to mission objectives.

#### Reporting Limits

Instrument reporting limits were determined by the range and precision of the sensors being used as provided by the manufacturers of the instrument sensors. This information is summarized in Table 2.

#### Accuracy

Accuracy measures how close results are to a true or expected value and can be determined by comparing a standard or reference sample to its actual value. According to the specifications of the SeapHOx and the CO_2_-Pro CV, the pH and *p*CO_2_ sensors have accuracies of ± 0.05 and 0.5% of the measured value, respectively. The Sea-Bird SeaFET and the SunBurst SAMI-CO_2_ sensors have accuracies of ± 0.05 and ± 0.3 μatm, respectively (Table 2). Due to the potential for signal drift and biofouling, the NEPs used laboratory-grade instruments to perform validations of autonomous data through measurement of discrete water samples collected from the instrument deployment sites. For example, in Tampa Bay, instrument sensors underwent laboratory validation in an instrument test tank which was cross-validated with discrete measurement of parameters. Accuracy of the measurements was ensured by using Certified Reference Materials for analysis of dissolved inorganic carbon (DIC) and total alkalinity (TA). Similar protocols were employed by the other NEPs. Discrete samples were typically collected every 1–6 weeks (see section “Discrete Sampling” for details).

#### Precision

The precision of data is a measure of the reproducibility of a measurement and includes components of random error. Precision is strictly defined as a measure of the closeness with which multiple analyses of a given sample agree with each other. For this reason, the most common method used to collect real-time data from pH and *p*CO_2_ sensors in the field by the NEPs was to average a minimum of five consecutive measurements made by the sensors at their respective maximum sampling frequencies. Should extreme variations occur, the sensors were checked to verify their proper functionality, and service was conducted depending on the nature of the problem.

#### Bias

Bias (or drift) is a measurement of correctness and includes components of systematic error. A measurement is considered unbiased when the value reported does not differ from the true value. For the SunBurst SAMI-CO_2_, internal periodic blanks were automatically run to correct for drift of the electro-optical system, while reference measurements of the light emitting diodes (LEDs) correct for interim deviations. Instruments were inspected, maintained, and cleaned periodically to contain drift caused by biofouling or other factors.

For Tampa Bay, the data were deemed biased if the sensor-collected values deviated from ± 0.05 pH unit for pH and ± 0.5 percent for *p*CO_2_. In Mission-Aransas Estuary, the data was deemed biased if the sensor collected values deviated from the specified accuracy values (i.e., ± 0.02 pH unit for pH and ± 3 μatm for *p*CO_2_).

### Discrete Sampling

In addition to *in situ* sensor measurements, discrete water samples were collected by the NEPs and their partners to validate the sensor measurements and provide additional analytical data necessary to characterize the water chemistry. These data are not included in the analysis for this study but will be the focus of forthcoming estuary-specific reports. The NEPs most often collected and analyzed discrete samples for pH, DIC, and TA. The frequency of discrete sample collection ranged from weekly (e.g., Barnegat Bay), to bi-weekly to monthly (e.g., Tillamook Bay; Tampa Bay; Mission-Aransas Estuary) to every 4–6 weeks (Casco Bay; San Francisco Bay) to quarterly (e.g., Santa Monica Bay) and was often timed to coincide with sensor cleaning, other maintenance, and data downloading.

Some NEPs also used discrete or *in situ* measurements collected by other research programs to cross-calibrate their sensor data. For example, Santa Monica Bay used conductivity, temperature, depth (CTD) profile data, collected quarterly by the Los Angeles County Sanitation District (LACSD) at nearby stations, to evaluate the comparability between those CTD measurements and the acidification mooring sensors.

In summary, sensor data collected across the NEPs was assured through factory and laboratory instrument calibrations, validation to water quality measurements (discrete samples), inspection of the data record for anomalous data, proper maintenance and examination for bias and fouling, checks against oceanographic properties and other programs’ data and electronic validation and verification. Only data obtained from *in situ* instrument sensors which were supported by appropriate quality control data and met the measurement performance specifications defined here were considered acceptable and used in this analysis.

In this manuscript, we focus on data QA/QC and validation derived from instrument resolution and limitations as provided by the manufacturers of the instrument sensors (Table 2) along with internal trend and correlation analysis (see section “Overall Patterns of *p*CO_2_ and pH”). Additional validation and continued refinement of protocols are underway across the NEPs as more data continues to be collected and will be the focus of their own forthcoming reports and publications.

### Descriptive Statistics

Statistical analyses of the data^3^ were performed using R Statistical Software (version 3.6.2) and Tableau Desktop (version 2020.2) data visualization software. As an initial assessment, the site-specific range, mean, and standard deviation of each measured parameter (*p*CO_2_, pH, temperature, salinity, and DO) were calculated for each northern hemisphere meteorological season: winter (December-February), spring (March-May), summer (June-August), fall (September-November). Box plots of monthly-binned data, multi-parameter time series plots, and cross-plots of measured parameters were prepared to visualize relationships among coastal carbonate system parameters within, and across the water bodies at short (diel) and longer (seasonal) time scales.

### Thermal and Nonthermal Controls on *p*CO_2_

Physical factors (e.g., temperature-dependent solubility, transport and mixing [including coastal upwelling and freshwater outflow]) and biological factors (e.g., primary production and respiration) are important controls on *p*CO_2_ changes in coastal and estuarine environments (DeGrandpre et al., 2002; Feely et al., 2008, 2018; Huang et al., 2015; Wanninkhof et al., 2015). To separate the solubility effect due to water temperature change on observed *p*CO_2_ (*p*CO_2*obs*_) and to contrast the effect of temperature with the cumulative influence of other physical and biological factors, we calculated (1) non-temperature controlled *p*CO_2_ values (*p*CO_2*bio/hydro*_) and (2) temperature-controlled *p*CO_2*thermal*_ values (*p*CO_2*thermal*_) following the approach of Takahashi et al. (2002). It is acknowledged that Takahashi et al. (2002) represents an open-ocean study with minimal salinity variance. The coastal systems discussed in this study experience large salinity swings, which will slightly impact Henry’s Law constant (K_*H*_), and, by extension, the dissolution of CO_2_ as described by Henry’s Law: ([CO_2*(aq)*_] = K_*H*_ * *p*CO_2_). *p*CO_2*bio/hydro*_ values were calculated for each water body on a site-specific basis by normalizing *p*CO_2*obs*_ values to the site-specific mean water temperature as calculated over the course of monitoring [Eq. (1)]:

(1) $$pCO_{2\;\text{bio}/\text{hydro}}=pCO_{2\;\text{obs}}\times e^{0.0423\left(T_{\text{mean}}-T_{\text{obs}}\right)}$$

where *pCO*_2*obs*_ is the *p*CO_2_ at i*n situ* temperature in degrees Celsius (°C), *T*_*mean*_ is the site-specific mean temperature, which ranged from 11.4°C in Tillamook to 24.2°C in Mission-Aransas, and *T*_*obs*_ is the site-specific *in situ* temperature in degrees Celsius. Changes in *p*CO_2*bio/hydro*_ primarily represent changes in *p*CO_2_ due to the combined influences of respiration and production, alkalinity, upwelling, diffusion of CO_2_ between the ocean and atmosphere, and advection past the sensor suite by tides and currents.

In contrast, to calculate the effect that only temperature would have on *p*CO_2*obs*_, we again followed the method of Takahashi et al. (2002):

(2) $$pCO_{2\;\text{thermal}}=pCO_{2\;\text{mean}}\times e^{0.0423\left(T_{\text{obs}}-T_{\text{mean}}\right)}$$

## RESULTS

### Overall Patterns of *p*CO_2_ and pH

**FIGURE 4 |:**
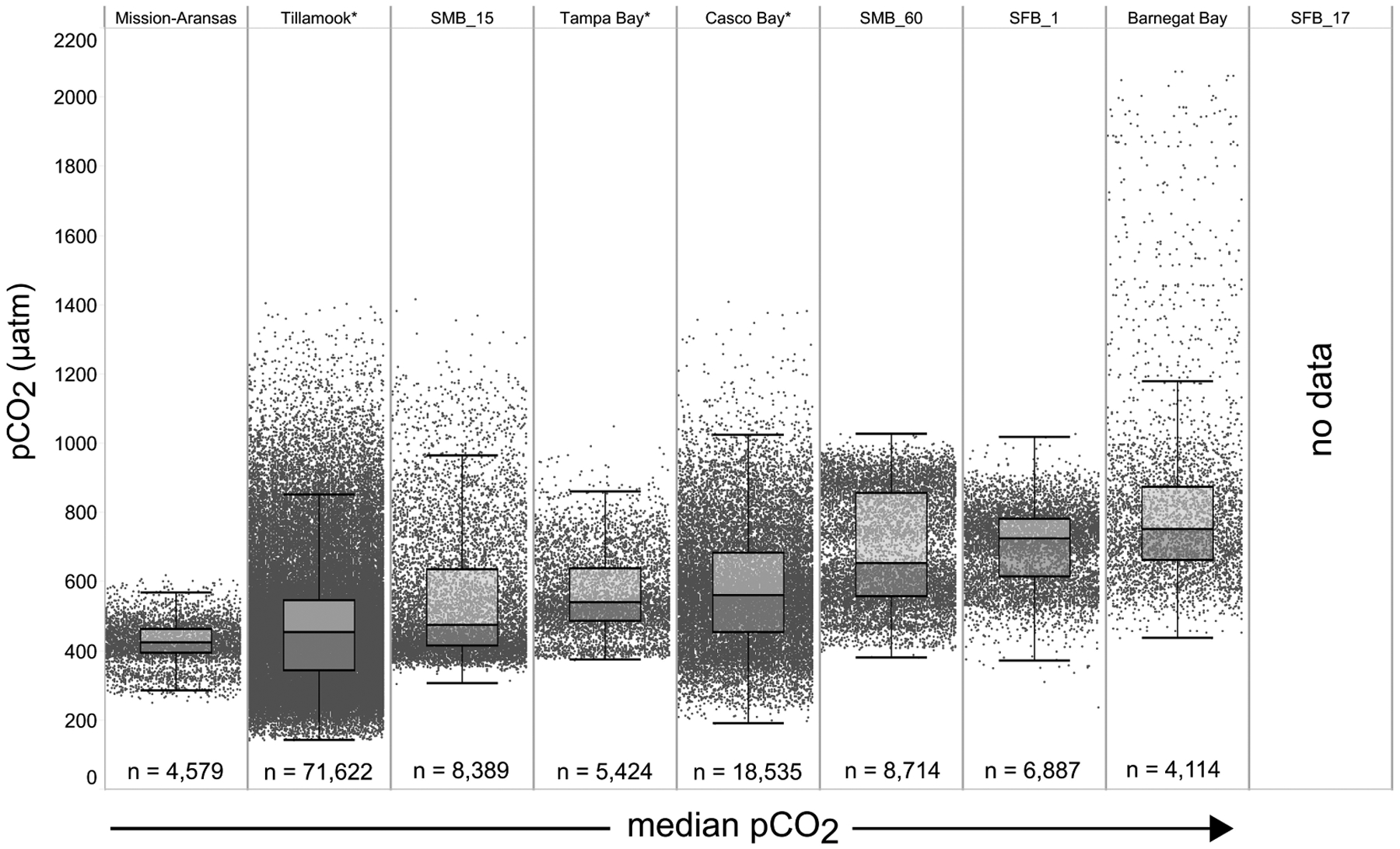
Box plots and underlying data showing the distribution of partial pressure of carbon dioxide (*p*CO_2_) data across the seven water bodies. The water bodies are arranged from lowest median *p*CO_2_ (Mission-Aransas Estuary) to highest median *p*CO_2_ (Barnegat Bay) as calculated from all available data. Whiskers extend to data within 1.5 times the interquartile range (IQR), * = multi-year records. SMB_15 = Santa Monica Bay (15 m deployment); SMB_60 = Santa Monica Bay (60 m deployment); SFB_1 = San Francisco Bay (1 m deployment). *p*CO_2_ data are not available for San Francisco Bay’s 17 m deployment.

**FIGURE 5 |:**
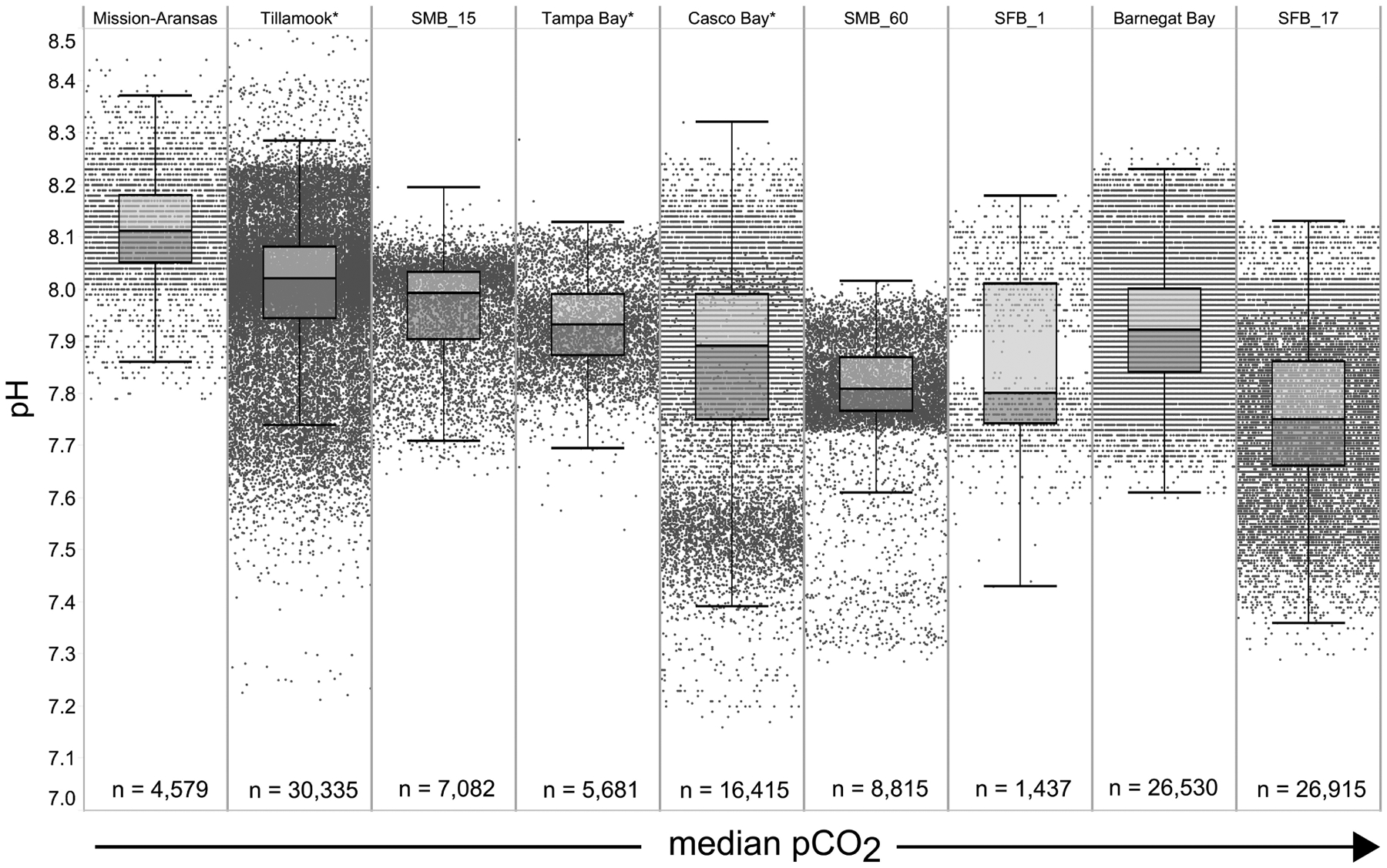
Box plots and underlying data showing the distribution of pH data across the seven water bodies. The water bodies are arranged from lowest median partial pressure of carbon dioxide (*p*CO_2_) (Mission-Aransas Estuary) to highest median *p*CO_2_ (Barnegat Bay) as calculated from all available data. Whiskers extend to data within 1.5 times the interquartile range; * = multi-year records. SMB. SMB_15 = Santa Monica Bay (15 m deployment); SMB_60 = Santa Monica Bay (60 m deployment); SFB_1 = San Francisco Bay (1 m deployment); SFB_17 = San Francisco Bay (17 m deployment).

Variability in observed *p*CO_2*obs*_ and pH are shown in Figures 4, 5. The mean, range, and standard deviation of the mean for each measured parameter on a seasonal basis are shown in Supplementary Table 3. Box plots with all underlying data shown illustrate the variability of *p*CO_2*obs*_ and pH data that were collected within and across the seven water bodies (Figures 4, 5). Variability discussed here refers to the difference between the upper and lower whiskers (1.5 times the interquartile range [IQR]) of the box plots. This was done to facilitate comparison of data across a range of systems with variable sample sizes and monitoring timelines and reduce the risk of including potentially short-lived, albeit likely real, extreme events. Note that all data included in the results and discussion passed Quality Assurance/Quality Control (QA/QC) checks and therefore are assumed to represent real data. The term “IQR *p*CO_2_” and “IQR pH” used in the following sections refers to the variability in *p*CO_2_ and pH values, respectively, that fall within 1.5 times the IQR.

The largest ranges of *p*CO_2_ values, based on the difference between the upper and lower whiskers of the box plots, were observed in Casco Bay (191 – 1,024 μatm) and Barnegat Bay (438 – 1,180 μatm; Figure 4). When all data are considered, Tillamook Bay has both the lowest (143 μatm) and highest (1,405 μatm) measured *p*CO_2_ values. In Casco Bay, variability in IQR *p*CO_2_ was associated with the correspondingly largest range in IQR pH (7.39 – 8.32) measured across all of the water bodies and highly variable salinity (Supplementary Figure 1). Similarly, in Barnegat Bay the variability in IQR *p*CO_2_ values was associated with a correspondingly large range in IQR pH (7.61 – 8.23) but smaller range of salinity values (Figures 4, 5, Supplementary Figure 1; Supplementary Table 3). The smallest range in IQR *p*CO_2_ was observed in Mission-Aransas Estuary (287 – 568 μatm), where IQR pH values ranged from 7.86 to 8.37 (Figures 4, 5). Ranges of IQR *p*CO_2_ (374 – 860 μatm) and pH (7.70 – 8.13) in Tampa Bay were similar to those in Mission-Aransas. The two Santa Monica Bay deployments exhibited the smallest ranges of temperature, salinity, and DO, with deep waters being the least variable (Figures 4, 5, Supplementary Figure 1; Supplementary Table 3). Deep waters in Santa Monica Bay also exhibited the smallest range of IQR pH values (7.61 – 8.01), although IQR *p*CO_2_ values were more variable (382 – 1,028 μatm; Figures 4, 5, Supplementary Table 3). Salinity values in both Santa Monica Bay deployments fell in a very narrow range (Supplementary Figure 1). Variability in IQR *p*CO_2_ and pH was greater in nearshore waters of Santa Monica Bay (Figures 4, 5). Variability in San Francisco Bay IQR *p*CO_2_ values (372 – 1,018 μatm) were similar to those observed in Santa Monica Bay. Deeper water Santa Monica Bay and San Francisco Bay showed a bimodal distribution of *p*CO_2_ values (Supplementary Figure 2). Time series plots of all measured parameters from all sites are shown in SupplementaryFigures2–6 and relationships between parameters are shown as cross-plots in Supplementary Figures 7–15. Frequency distribution plots for *p*CO_2_ and pH data from each water body are shown in Supplementary Figures 16–17.

### Seasonal Patterns in *p*CO_2_ and pH

**FIGURE 6 |:**
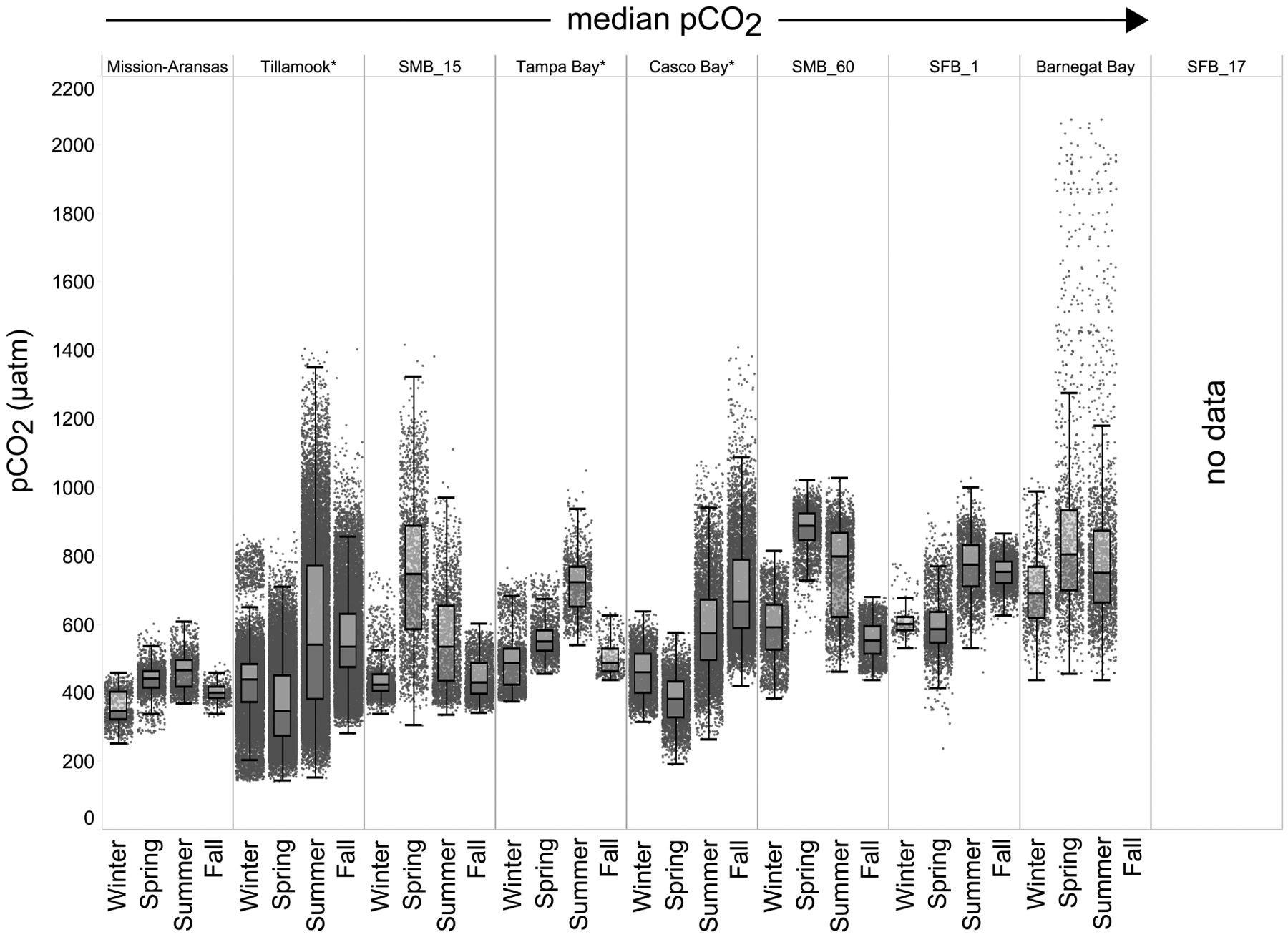
Box plots and underlying data showing the seasonal distribution of partial pressure of carbon dioxide (*p*CO_2_), data across the seven water bodies. The water bodies are arranged from lowest median *p*CO_2_ to highest median *p*CO_2_. Whiskers extend to data within 1.5 times the interquartile range (IQR). Northern hemisphere meteorological season (winter – December, January, February; spring – March, April, May; summer – June, July, August; fall – September, October, November. * = multi-year records. SMB_15 = Santa Monica Bay (15 m deployment); SMB_60 = Santa Monica Bay (60 m deployment); SFB_1 = San Francisco Bay (1 m deployment). *p*CO_2_ data are not available for the San Francisco Bay 17 m deployment.

**FIGURE 7 |:**
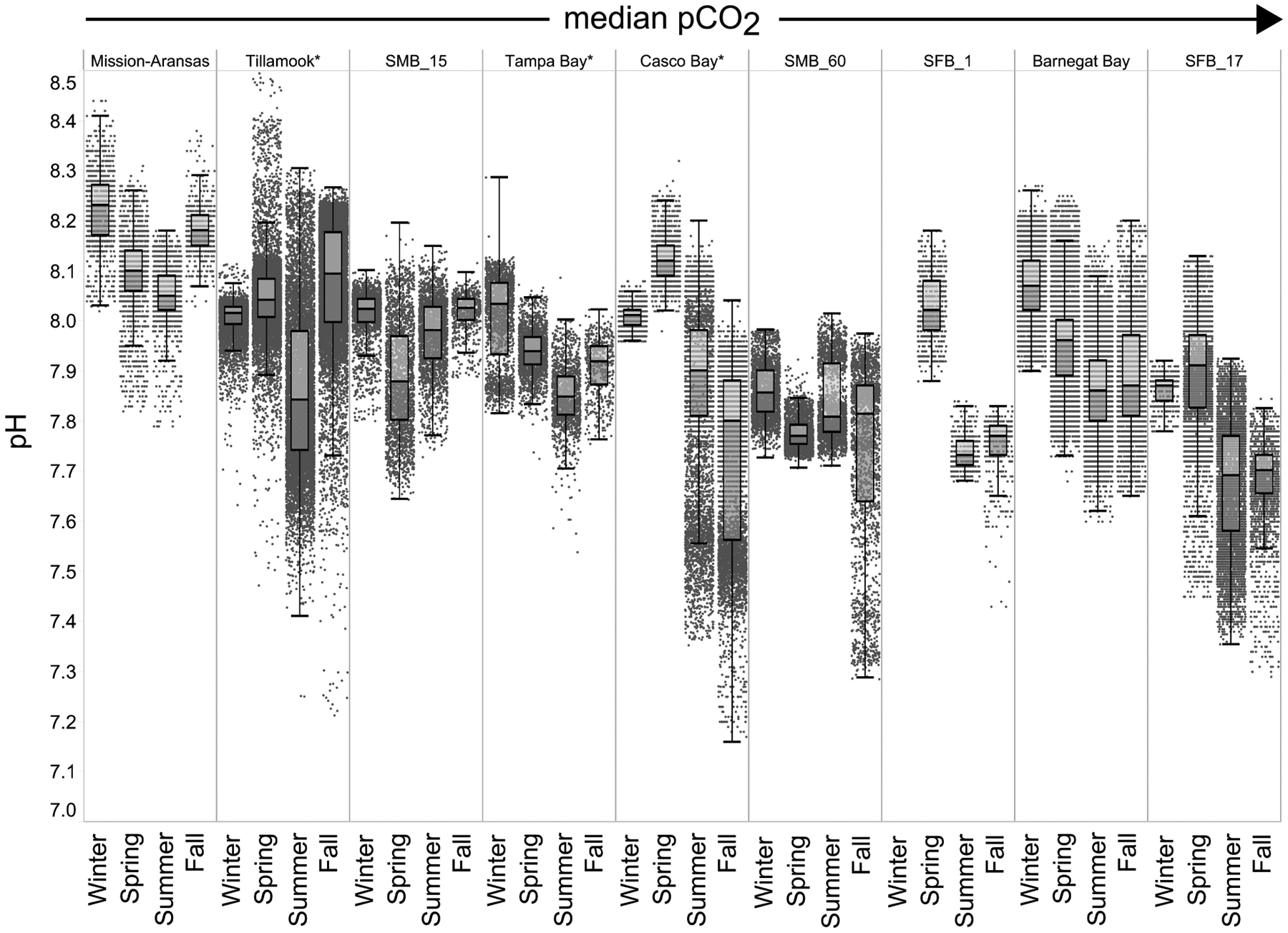
Box plots and underlying data showing the seasonal distribution of pH data across the seven water bodies. The water bodies are arranged from lowest median partial pressure of carbon dioxide (*p*CO_2_) to highest median *p*CO_2_. Whiskers extend to data within 1.5 times the interquartile range (IQR). Northern hemisphere meteorological season (winter – December, January, February; spring – March, April, May; summer – June, July, August; fall – September, October, November. * = multi-year records. SMB_15 = Santa Monica Bay (15 m deployment); SMB_60 = Santa Monica Bay (60 m deployment); SFB_1 = San Francisco Bay (1 m deployment); SFB_17 = San Francisco Bay (17 m deployment).

**FIGURE 8 |:**
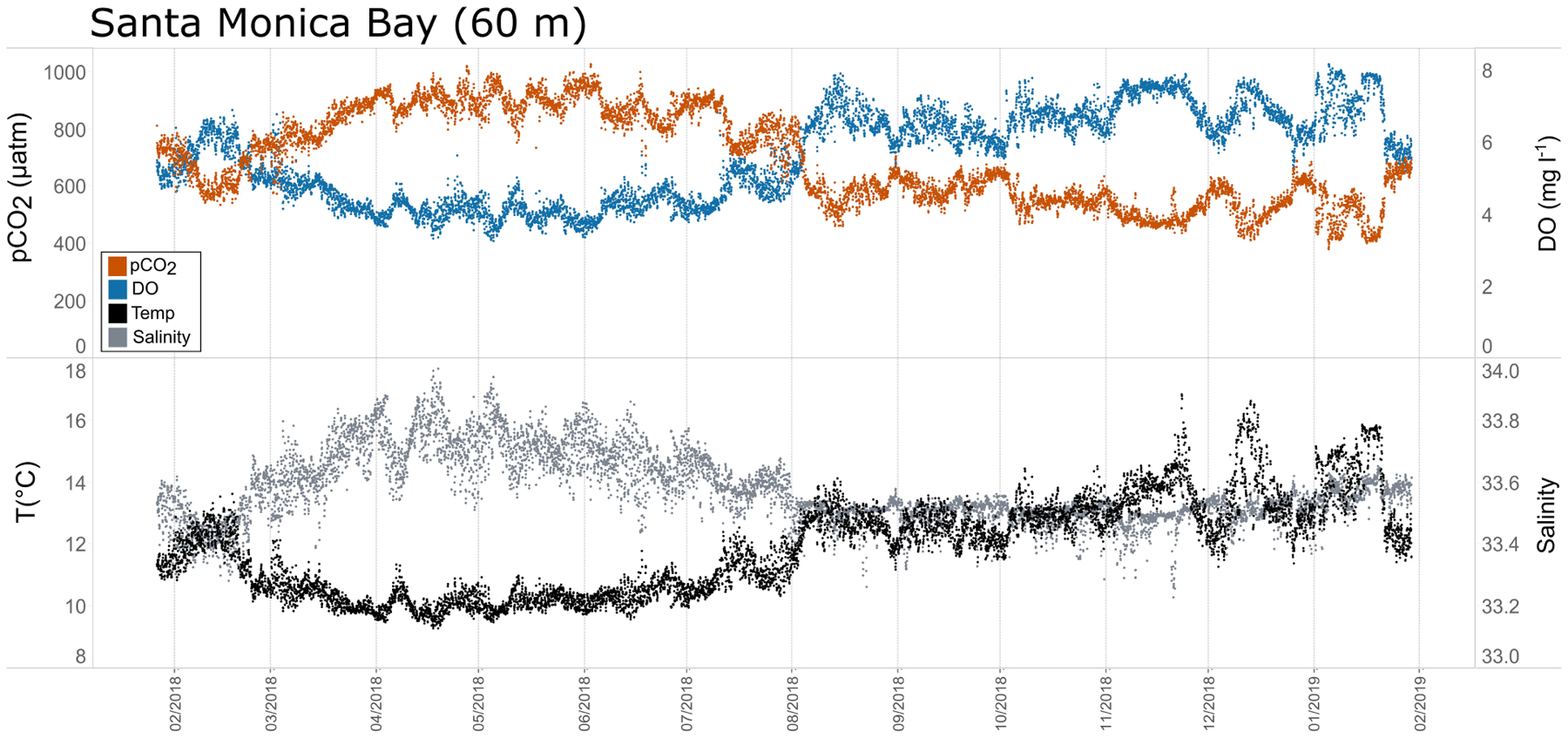
Time series plots of partial pressure of carbon dioxide (*p*CO_2_), dissolved oxygen (DO), temperature, and salinity in Santa Monica Bay (60 m; January 2018–February 2019.

**FIGURE 9 |:**
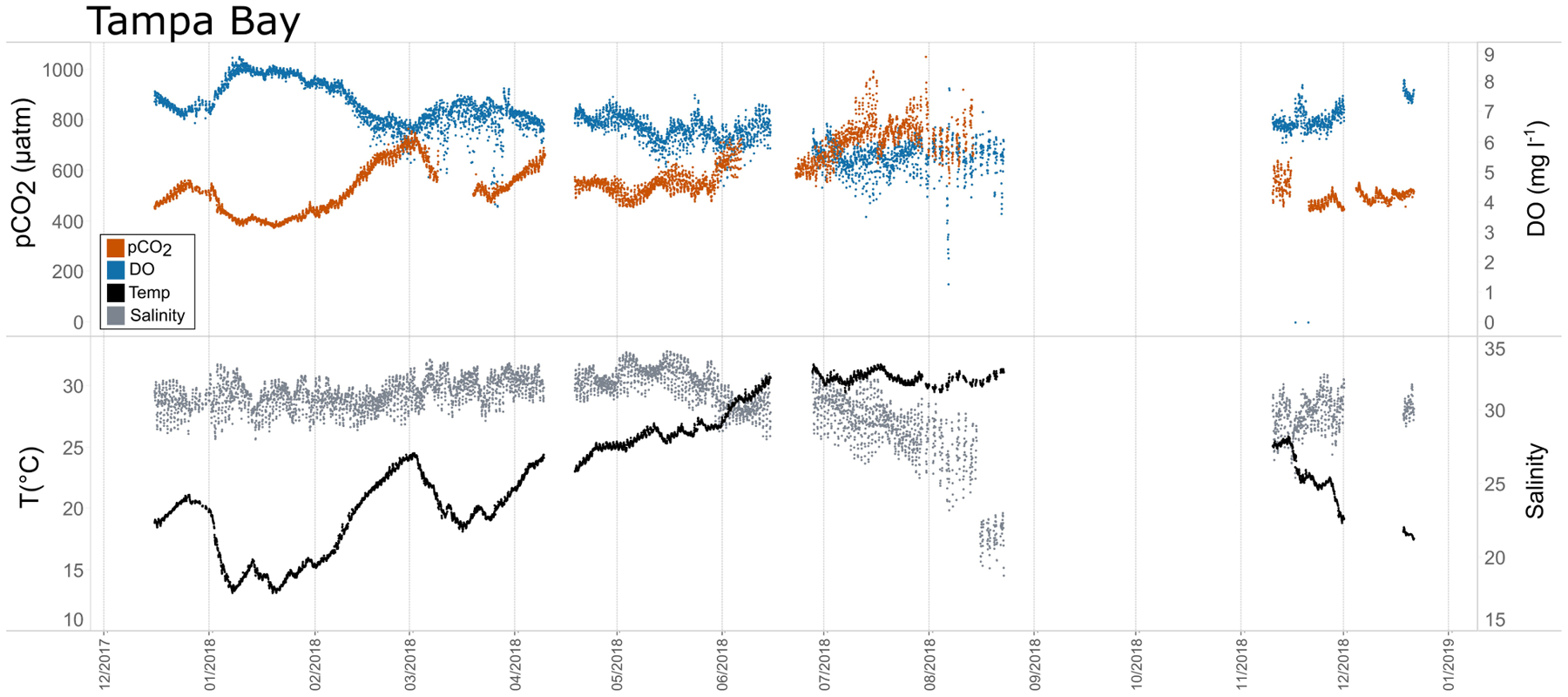
Time series plots of partial pressure of carbon dioxide (*p*CO_2_), dissolved oxygen (DO), temperature, and salinity in Tampa Bay (December 2017–January 2019).

**FIGURE 10 |:**
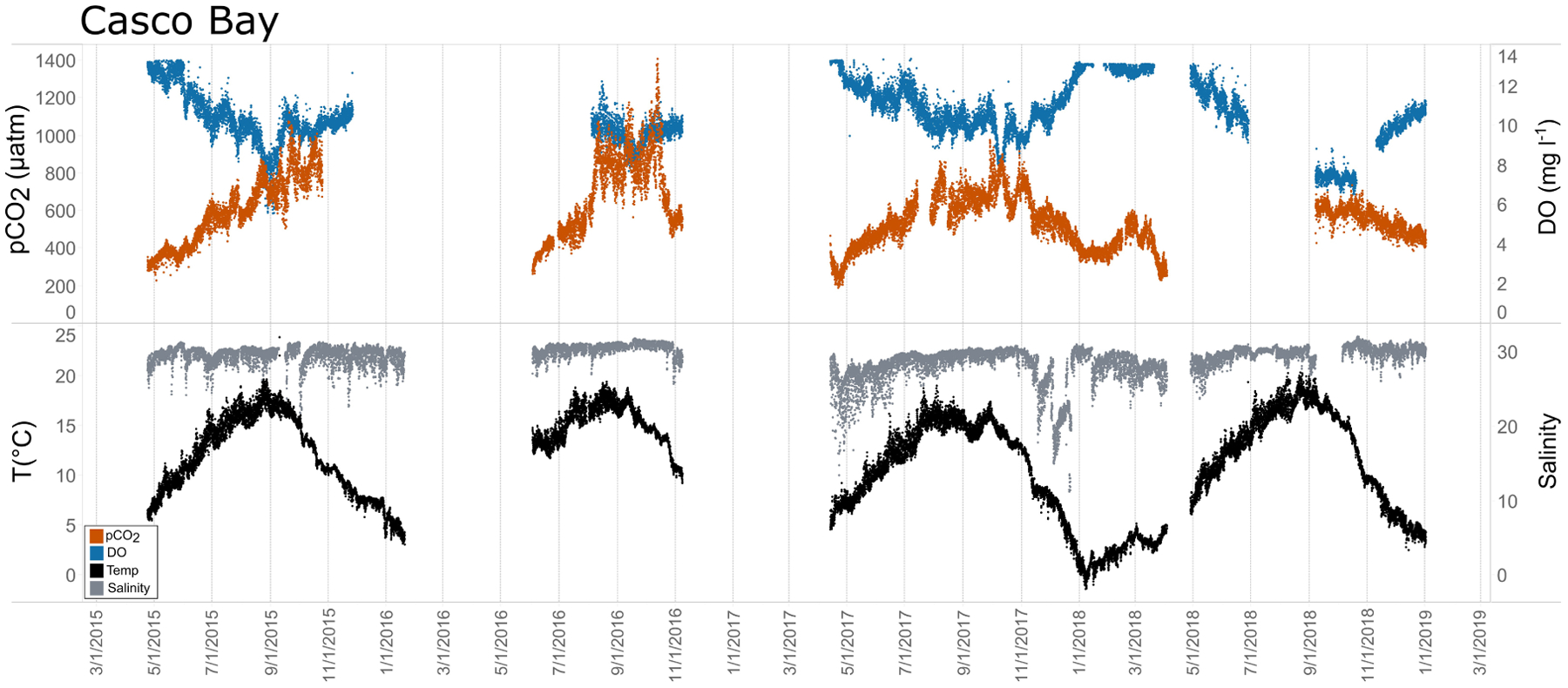
Time series plots of partial pressure of carbon dioxide (*p*CO_2_), dissolved oxygen (DO), temperature, and salinity in Casco Bay (April 2015–December 2018).

**FIGURE 11 |:**
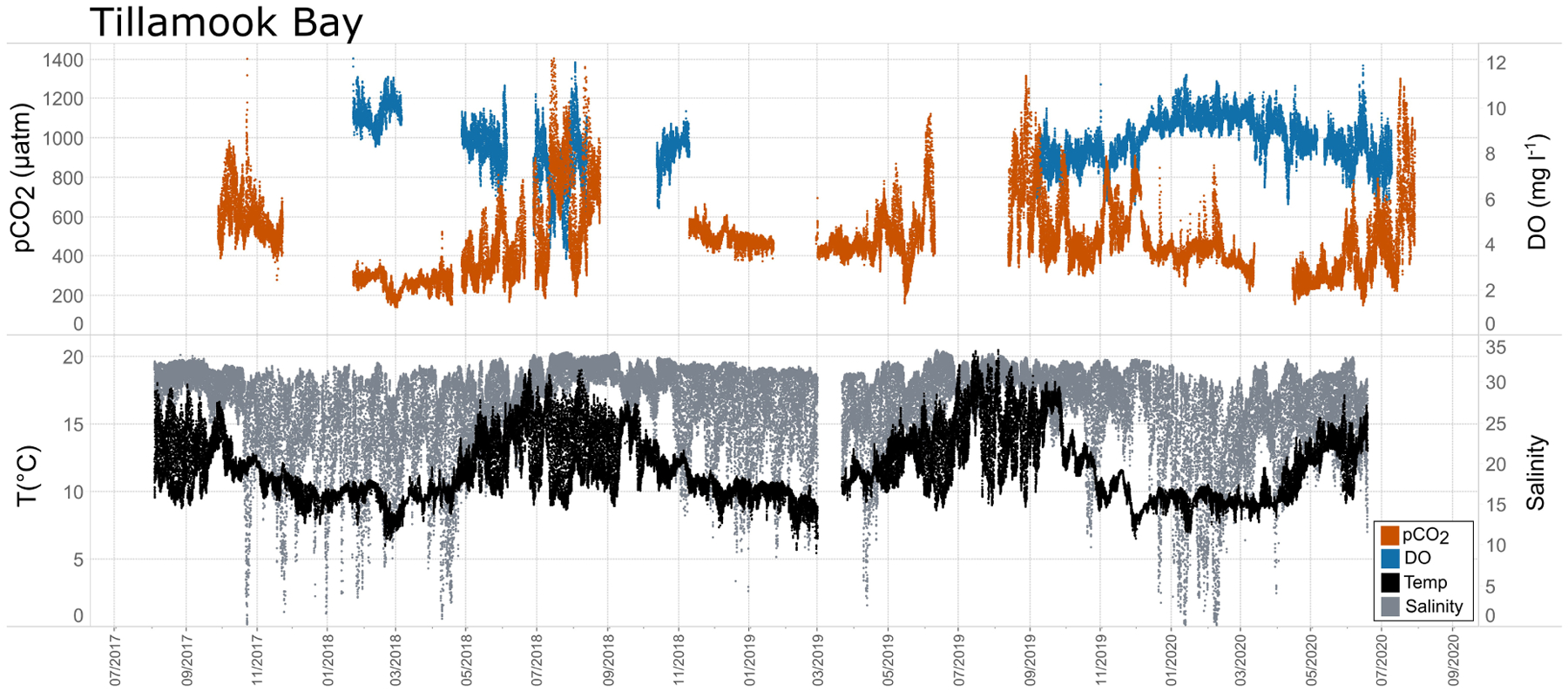
Time series plots of partial pressure of carbon dioxide (*p*CO_2_), dissolved oxygen (DO), temperature, and salinity in Tillamook Bay (July 2017–July 2020).

Seasonal patterns in *p*CO_2_, pH, temperature, salinity, and DO were observed across the water bodies. In general, *p*CO_2_ values were higher and more variable in warmer, summer months and lower and less variable during cooler, winter months (Figures 6–11; Supplementary Figure. 2). The major exception to this pattern is Santa Monica Bay, where the highest *p*CO_2_ values were observed during the spring (Figures 6, 8; Supplementary Figure 2). Both Mission-Aransas Estuary and Tampa Bay displayed a pattern of increasing *p*CO_2_ from cooler, winter months into warmer, summer months with yearly *p*CO_2_ values reaching their peak in July and August (Figures 6, 9). Overall, the magnitude of these seasonal changes in *p*CO_2_ is significantly muted in Mission-Aransas Estuary and Tampa Bay as compared to the other water bodies. Salinity was more variable throughout the year in Mission-Aransas than in Tampa Bay (Supplementary Figure 18). Similar seasonal patterns of *p*CO_2_ variability were observed in Casco Bay and Tillamook Bay where data from seasons over consecutive years are available (Figures 6, 10, 11, Supplementary Figures 2, 3). In these water bodies, however, a shift towards a trend of increasing *p*CO_2_ values began earlier in the year- in late winter (February) in Tillamook Bay and in mid-spring (April) in Casco Bay- with maximum *p*CO_2_ values generally occurring in late summer-early fall. A shift back towards decreasing *p*CO_2_ values occurred in mid-summer (August) in Tillamook Bay to mid-fall (October) in Casco Bay. These seasonal patterns and the relative magnitude of seasonal variability are comparable across consecutive years [i.e., 2015–2018 (Casco Bay); 2017–2019 (Tillamook Bay)]; although, 2019 winter – early Spring *p*CO_2_ values in Tillamook Bay were slightly elevated as compared to 2018 winter values. In Santa Monica Bay, *p*CO_2_ values increased from the winter into the spring with values reaching their maximum in May before steadily decreasing through the fall and reaching their lowest values during the winter (Figures 6, 8). High *p*CO_2_ values in the spring-early summer in Santa Monica Bay are associated with high salinity, low temperature, low DO water (Supplementary Figure 18; Figure 8), indicative of waters that have upwelled during the spring and early summer. Similar patterns of seasonal *p*CO_2_ variability were observed in San Francisco Bay; however, the shift from decreasing to increasing *p*CO_2_ values occurred later in spring (~April). During the summer, *p*CO_2_ values were highest and remained relatively high (monthly median ~720–750 ppm) through December. *p*CO_2_ data for Barnegat Bay is relatively sparse compared to other water bodies and there is variability in the data; however, there are indications of a similar trend of increasing *p*CO_2_ from winter months into the spring; however, there is variability in the data (Figure 6).

In general, seasonal trends in pH were opposite of those observed in *p*CO_2_ throughout the year with rises and falls in *p*CO_2_ associated with falls and rises, respectively, in pH (Figure 7). Significant variability (i.e., large range of values) in pH was observed throughout the year in all water bodies, but this variability was particularly large in summer and fall months where it was observed across multiple connective years in Tillamook Bay and Casco Bay, respectively (Figures 7, 10, 11). Some of the lowest recorded pH values across all of the water bodies were associated with these periods of high variability in pH and salinity values in Tillamook and Casco bays (Figure 7; Supplementary Figures 3, 18). Deeper water in Santa Monica Bay and San Francisco Bay showed similarly low pH values in the fall and summer, respectively (Figure 7). Overall, Mission-Aransas Estuary, Tampa Bay, and Barnegat Bay share similar patterns of seasonal pH variability with pH values generally falling from the winter into the summer followed by a trend towards higher values in the fall and back into the winter (Figure 7). The deep and shallow records from Santa Monica Bay show similar seasonal patterns of pH with values decreasing from the winter into the spring and then generally increasing into the fall, a pattern opposite of that observed for *p*CO_2_ (Figures 6, 7). Although *p*CO_2_ variability in Santa Monica Bay and San Francisco Bay was quite similar, seasonal patterns of pH variability within these two water bodies were very different. In San Francisco Bay, pH values were highest in spring followed by an overall decrease in pH values through the fall before a return to slightly more positive values in the winter (Figure 7). This pattern was particularly clear at the 17 m sensor in San Francisco Bay, where a more complete dataset exists.

### Thermal and Nonthermal *p*CO_2_ Calculations

**FIGURE 12 |:**
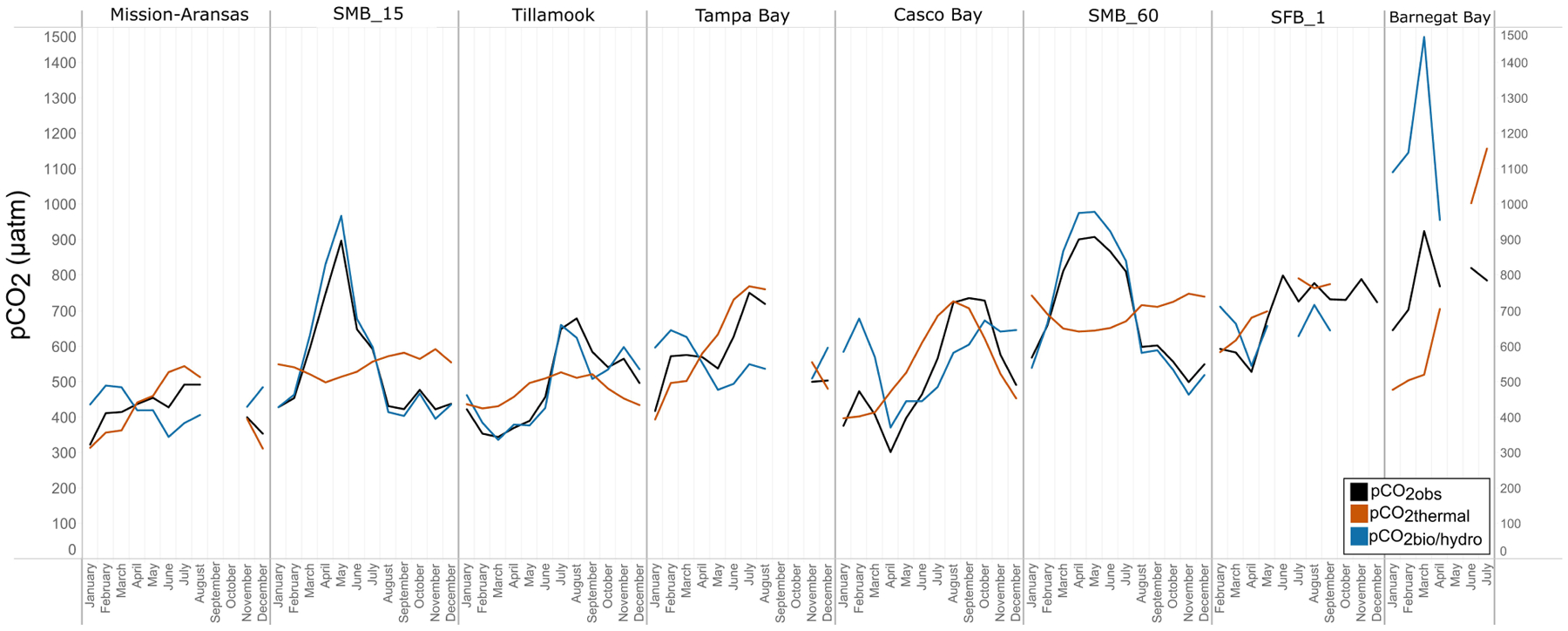
Trends in *p*CO_2*obs*_, *p*CO_2*thermal*_, and *p*CO_2*bio*/*hydro*_ within in each water body. Data points represent monthly averages. Black line = *p*CO_2*obs*_; red = *p*CO_2*thermal*_; blue = *p*CO_2*bio*/*hydro*_. The water bodies are arranged from lowest median *p*CO_2_ to highest median *p*CO_2_.

Calculations of thermal and nonthermal controls on *p*CO_2_, referred to as *p*CO_2*thermal*_ and *p*CO_2*bio/hydro*_, respectively, were made in order to contrast the influence of water temperature on *p*CO_2_ variation and the aggregate of other processes such as primary productivity, respiration, and mixing. These theoretical *p*CO_2_ values and *in situ*, observed *p*CO_2_ values (*p*CO_2*obs*_) are shown in Figure 12. Plots of all *p*CO_2*obs*_, *p*CO_2*thermal*_ and *p*CO_2*bio/hydro*_ data are in Supplementary Figures 19–26. Relationships and patterns of *p*CO_2*obs*_, *p*CO_2*thermal*_ and *p*CO_2*bio/hydro*_ are similar in Tampa Bay and Mission-Aransas Estuary. A trend towards increasingly higher *p*CO_2*thermal*_ values was observed from the winter through summer months in these GOM water bodies, with peak *p*CO_2*thermal*_ values occurring in July (Figure 12). In general, seasonal patterns of *p*CO_2*bio/hydro*_ are opposite of those seen in *p*CO_2*thermal*_, with *p*CO_2*bio/hydro*_ values falling from the winter through summer months and reaching their lowest values in the summer (Figure 12).

In Casco Bay, *p*CO_2*obs*_, *p*CO_2*thermal*_, and *p*CO_2*bio/hydro*_ showed broadly similar patterns throughout the year, and seasonally across consecutive years (Figure 12, Supplementary Figure 24). During the spring, *p*CO_2*obs*_ values were well below what would be expected from temperature alone (*p*CO_2*thermal*_). *p*CO_2*obs*_ values generally increased from spring into the fall, reaching their highest values around October, before beginning to decrease into the cooler, fall and winter months. *p*CO_2*bio/hydro*_ values continued to increase into the late fall, reaching their maximum around October, while *p*CO_2*thermal*_ values reached their maximum around August before beginning to decrease into the cooler, winter months. Only a few months of data are available for Barnegat Bay, therefore seasonal patterns were not distinguished (Figure 12).

Relationships among *p*CO_2*obs*_, *p*CO_2*thermal*_, and *p*CO_2*bio/hydro*_ and seasonal patterns of *p*CO_2_ variability are very different in West Coast water bodies. In Santa Monica and Tillamook bays, *p*CO_2*bio/hydro*_ was more closely associated with *p*CO_2*obs*_ while seasonal undulations in *p*CO_2*thermal*_ were less extreme, as compared to the patterns in Mission-Aransas Estuary and Tampa Bay (Figure 12; Supplementary Figures 19, 20, 22). Although muted, *p*CO_2*thermal*_ values in Santa Monica Bay (both depths/records), generally decreased from winter through spring. This is followed by an upward trend towards more positive *p*CO_2*thermal*_ values from spring into summer. Seasonal patterns in *p*CO_2*bio/hydro*_ were similar across these three water bodies (Figure 12). At both depths in Santa Monica Bay and in Tillamook Bay, *p*CO_2*bio/hydro*_ and *p*CO_2*obs*_ tracked one another very closely throughout the year (i.e., there was little influence of changes in solubility associated with changes in temperature). In Tillamook, this pattern was observed over multiple consecutive years.

### Diel Patterns in *p*CO_2_

**FIGURE 13 |:**
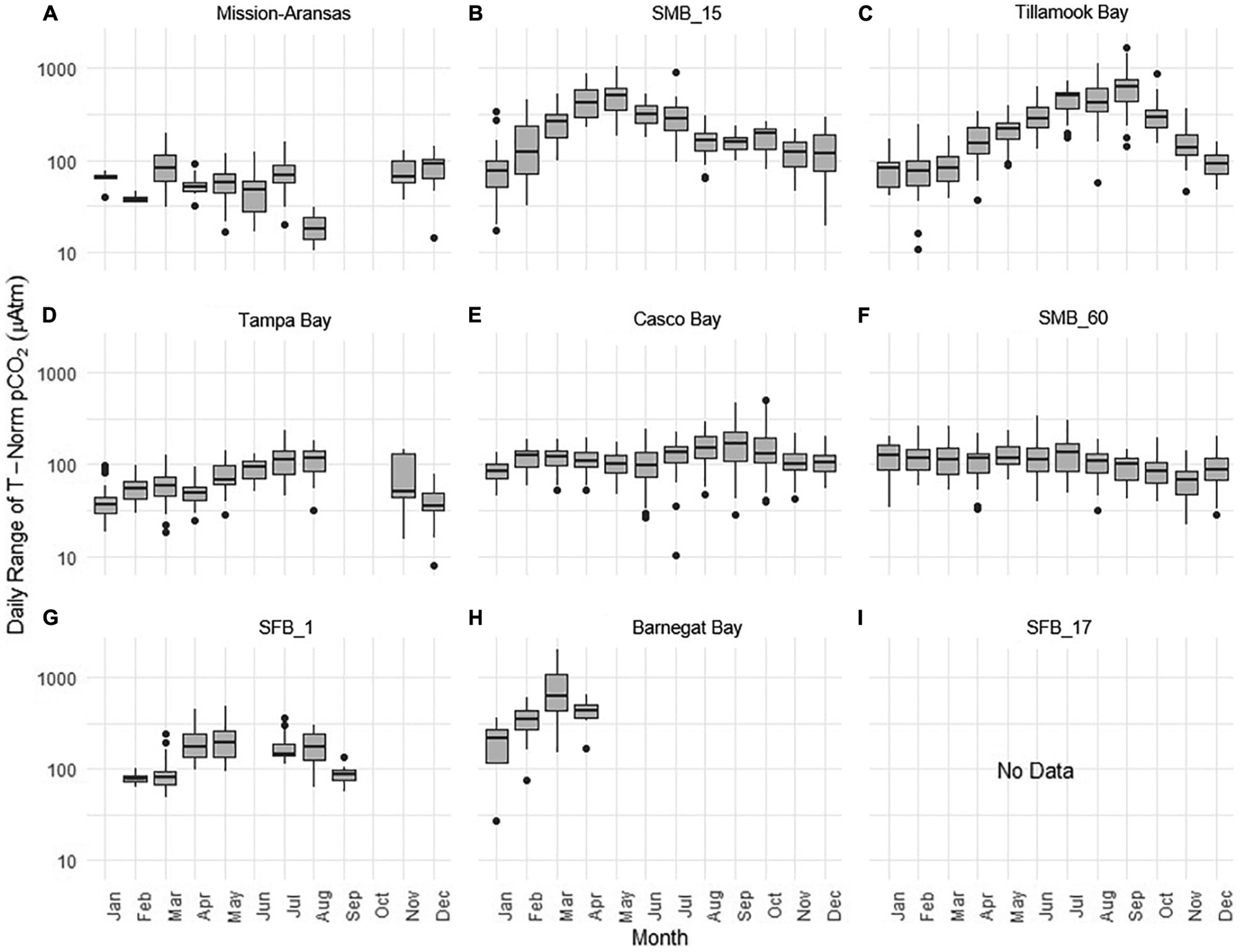
Box plots highlighting diel range of partial pressure of carbon dioxide (*p*CO_2_) *p*CO_2*bio*/*hydro*_. The water bodies are arranged from lowest median *p*CO_2_ to highest median *p*CO_2_: **(A)** Mission-Aransas Estuary; **(B)** Santa Monica Bay (SMB_15); **(C)** Tillamook Bay; **(D)** Tampa Bay; **(E)** Casco Bay; **(F)** Santa Monica Bay (SMB_60); **(G)** San Francisco Bay (SFB_1); **(H)** Barnegat Bay; **(I)** San Francisco Bay (SFB_17). Data binned by month. *Y*-axis is log-10 scale.

Daily ranges of monthly binned nonthermal *p*CO_2_ (*p*CO_2*bio/hydro*_) values binned by month are expressed in box plots and highlight the extent to which diel ranges vary throughout the year within, and across the water bodies (Figure 13). In general, with the exception of Santa Monica Bay and San Francisco Bay, overall patterns are similar to those observed in the monthly binned data in that there is greater diel variability in warmer months. Some of the greatest diel fluctuations in *p*CO_2*bio/hydro*_ were observed in Tillamook Bay during the late summer–early fall with values increasing from the spring into fall (Figure 13C). Large diel ranges in *p*CO_2*bio/hydro*_ were observed at nearshore Santa Monica Bay and near-surface San Francisco Bay where diel variability increased through spring and summer and the largest ranges were found in early summer (Figures 13B,G). Consistent and relatively small diel ranges in *p*CO_2*bio/hydro*_ were observed in deeper waters in Santa Monica Bay, which is below the euphotic zone (Figure 13F). Large diel ranges were observed in Barnegat Bay as well; however, data are only available for January–April (Figure 13H). Overall, diel variability in *p*CO_2*bio/hydro*_ in Tampa Bay and Mission-Aransas Estuary was smaller compared to the other water bodies (Figures 13A,D). Median ranges were typically < 100 μatm in Mission-Aransas Bay and Tampa Bay throughout the year.

## DISCUSSION

Estuaries and coastal waters are highly vulnerable to the impacts of acidification. Yet, little is known about the extent of this vulnerability and the relative influence of estuary-specific drivers that contribute to acidification, such as intrusion of CO_2_-rich seawater, nutrient enrichment, locally elevated atmospheric CO_2_ from urban and agricultural activities, net ecosystem production and respiration, and freshwater flows (Salisbury et al., 2008; Feely et al., 2008, 2010, 2018; Gledhill et al., 2015; Northcott et al., 2019; Rheuban et al., 2019). This multitude of influences leads to greater pH and *p*CO_2_ variability and more acidic conditions as compared to open ocean values (Cai et al., 2021). For example, pH values ranged from 7.16 to 8.32 in Casco Bay and from 7.21 to 8.31 and in Tillamook Bay whereas the global range of surface ocean pH values is ~7.7 to 8.7 (Takahashi et al., 2014; Bakker et al., 2016; Jiang et al., 2019). Comparably large pH ranges, up to 1.2 within a single month, have been observed at other coastal sites with some of the most extreme pH values showing large interannual variability (e.g., Dorey et al., 2013; Sutton et al., 2016; Pecquet et al., 2017). Autonomous surface deployments in coastal settings have recorded seasonal (winter-summer) CO_2_ differences of up to 330 μatm compared to difference up to 70 μatm in open ocean settings (Shadwick et al., 2015; Sutton et al., 2019). For comparison, seasonal *p*CO_2_ differences observed in the coastal systems described here are ~2–4x as large (e.g., Casco Bay – 833 μatm; Barnegat Bay – 697 μatm; Santa Monica Bay – 646 μatm), the largest being observed in Tillamook Bay (1,263 μatm). In some cases (e.g., Tillamook Bay, Casco Bay, Santa Monica Bay_15 m), diel amplitudes in *p*CO_2_ and pH are comparable to those observed seasonally. Similar observations have been made in other coastal systems (Torres et al., 2021), albeit the amplitudes were not as extreme as those observed in this study. Given the variability in estuarine systems, multi-year to decadal deployments will be needed to more fully understand the natural variability and important processes driving acidification in these systems. For this initial data synthesis, analyses were restricted to shorter (e.g., seasonal) time scales that are appropriate for existing datasets.

An important goal of the NEP coastal acidification monitoring program is to inform longer-term management decisions as more data are collected. For example, aragonite saturation state (ΩAr) is an important calculated measure that is most closely related to organism health for calcifiers and can be used to understand the vulnerability of water bodies to acidification. This comparative study did not calculate ΩAr for each water body because additional parameterization of the carbonate chemistry, including validation data, is necessary and was not available on a consistent basis across the water bodies. Specifically, calculating aragonite saturation requires that, in addition to temperature and salinity, at least two of the carbonate parameters (*p*CO_2_, total alkalinity, DIC, pH) be known. However, *p*CO2 and pH are not an ideal set of input parameters for calculating ΩAr because they carry the largest uncertainties compared to other input pairs (Millero, 1995; Orr et al., 2018) and the controls on these parameters (e.g., pH and ΩAr) are not entirely consistent (Xue et al., 2021). Such uncertainties may be even larger for estuarine sites with highly variable salinity as disassociation constants are poorly constrained compared with ocean salinity. Ideally a better constrained ΩAr dataset should be independently verified through inclusion of additional CO_2_ system parameters (e.g., DIC and TA). However, these data were not collected across all water bodies, and as such, the calculated ΩAr results would be based on inconsistent methodologies (i.e., using variable input parameters).

Rather, this study’s focus is on patterns of *p*CO_2_ and pH variability and the relationship of these parameters to temperature, salinity, and DO. A similar preliminary analysis of high-frequency monitoring data from the National Estuarine Research Reserve System has shown concurrent fluctuations in pH and DO to be characteristic of nearshore habitats and explored how pH data paired with DO data could be recording the effects of enhanced coastal acidification (Baumann and Smith, 2017).

### Patterns of *p*CO_2_ and pH

In general, across all water bodies, temperature and *p*CO_2_ were lower in cooler, winter months and higher in warmer, summer months. A similar pattern has been observed in other coastal systems (Wallace et al., 2014).

Higher-latitude, cooler water bodies like Tillamook Bay, Casco Bay, and Barnegat Bay and the deeper waters of California coastal water bodies have slightly higher *p*CO_2_ and lower pH than warmer GOM water bodies. This is likely due in part to the large annual temperature variability that typifies these higher latitude systems as compared to subtropical, warmer GOM systems (Tampa Bay and Mission-Aransas). For example, in Barnegat Bay in the northeastern United States, there was a 31°C range in annual water temperature (−1.9°C to 29.3°C) while in Tampa Bay in the GOM, the annual temperature range was 19°C (13.1°C to 31.8°C). The higher *p*CO_2_ values observed in Santa Monica Bay and San Francisco Bay are best explained by the upwelling of cold, high *p*CO_2_, low pH water rather than a large annual temperature range; annual temperatures in the deeper water Santa Monica Bay and San Francisco Bay deployments only vary by ~11.6°C. Furthermore, the timing of shifts toward increasing (or decreasing) *p*CO_2_ values varied by location and appear to be largely dictated by local climate conditions, leading to increases in *p*CO_2_ that began earlier in the year in the warmer water, lower latitude GOM water bodies of Tampa Bay and Mission-Aransas Estuary as compared to cooler water, higher latitude water bodies such as Tillamook Bay and Casco Bay. Thermally driven changes are also inferred to be largely responsible for the lower variability of *p*CO_2_ values observed in lower latitude, warmer water bodies as compared to higher latitude, cooler water bodies that have larger ranges of annual temperature variability.

Strong seasonal variations in *p*CO_2_ have been observed in other coastal systems (see Jiang et al., 2021 for a recent compilation of coastal data sets). For example, in the Delaware Bay, during the warmer summer months, *p*CO_2_ values of ~400–500 μatm, have been recorded, with values reaching 200 to 350 μatm in the mid- and lower bay regions, while in the winter (December) *p*CO_2_ measured across the bay ranged from 500–650 μatm (Joesoef et al., 2015). Biological and thermal controls as well as variable mixing and stratification and variability in river discharge have been inferred as the dominant controls responsible for this variability (Joesoef et al., 2015).

Other coastal systems in the northeastern United States (e.g., Long Island Sound, NY, United States; Narraganset Bay, RI, United States; Jamaica Bay, NY, United States; Hempstead Bay, NY, United States) are characterized by high *p*CO_2_ (>1000 μatm) coupled with low DO and low pH (<7.4) during warmer summer months (Wallace et al., 2014). These patterns have been largely attributed to high rates of microbial respiration driven by enhanced nutrient loadings coupled with water column stratification with changes in summer temperature accounting for < 5% of the *p*CO_2_ increases (Wallace et al., 2014).

Sensor deployment location in the estuary (nearshore, shallower water vs. offshore, deeper water) surely impacted the data collected during this study via the extent to which various processes are at play (e.g., freshwater runoff, biological activity, tides, heat dissipation [shallow vs. deep]) in these different locations. This will be and important consideration in future efforts to tease out drivers of acidification in these water bodies and to facilitate comparison with other data sets.

### Thermal and Nonthermal *p*CO_2_ Calculations

In addition to seasonal changes in temperature, the *p*CO_2_ in near-surface waters that exchange CO_2_ directly with the atmosphere is affected by DIC, pH and alkalinity. Water temperature is primarily regulated by physical processes (i.e., solar energy input, ocean-atmosphere heat exchange, and upwelling). DIC concentration and alkalinity, however, are controlled by a combination of physical (transport and mixing of different water masses, air-sea gas exchange) and biological processes (i.e., photosynthesis and respiration; carbonate precipitation and dissolution) (Feely et al., 2008, 2018; Cai et al., 2020).

To better understand the relative influence of water temperature with respect to other physical and biological processes, theoretical *p*CO_2*thermal*_ and non-thermal *p*CO_2*bio/hydro*_ values were calculated. Respectively, these values reflect the predicted *p*CO_2_ values if temperature were the sole control on *p*CO_2_ and the predicted *p*CO_2_ in the absence of temperature change. Changes in *p*CO_2*bio/hydro*_ primarily represent changes in total CO_2_ due to the combined influences of biology and diffusion of CO_2_ between the ocean and atmosphere, as well as advection past the sensor suite by tides and currents in the absence of temperature changes. Changes in *p*CO_2*thermal*_ are theoretical changes in *p*CO_2_ as a result of the decreasing solubility of CO_2_ with increasing temperature. Therefore, the relative magnitudes of *p*CO_2*thermal*_ (Δ*p*CO_2*thermal*_) and *p*CO_2*bio/hydro*_ (Δ*p*CO_2*bio/hydro*_) should reflect the dominant factor (i.e., temperature vs. other parameters) in controlling the observed *p*CO_2_ changes throughout these individual monitoring periods. The overall pattern across the estuaries is that Δ*p*CO_2*thermal*_ is smaller compared to the magnitude of *p*CO_2*bio/hydro*_ except in Mission-Aransas Estuary and Tampa Bay. This is consistent with a previously well-described pattern of temperature controlling open-ocean surface-water seasonal *p*CO_2_ variations at subtropical and lower temperate latitudes and biophysical processes exerting more significant control at moderate and higher latitudes (Takahashi et al., 2002).

Collectively, the observed differences among *p*CO_2*thermal*_, *p*CO_2*bio/hydro*_, and *p*CO_2*obs*_ values described here indicate that temperature and the aggregate of other factors, including seasonal net community productivity, tidal fluctuations, and freshwater inflows are equally important in controlling *p*CO_2_ variability in Mission-Aransas and Tampa Bay with the degree to which these processes impact *p*CO_2*obs*_ varying throughout the year. In Tampa Bay, strong tidal control and the location of the sensor package near the mouth of the bay may have resulted in capturing of variable and complex signatures due to both water mass exchange between Tampa Bay and the GOM and associated salinity and DO differences and biological productivity.

Multidecadal decreases in both total alkalinity and pH have been observed in Mission-Aransas Estuary (Hu et al., 2015). The rivers feeding Mission-Aransas Estuary are highly alkaline and the long-term acidification has consequently been attributed to decreased freshwater inflow (and a resulting decreased alkalinity delivery) to the estuary due to freshwater diversions (Hu et al., 2015). The relative importance of thermal and biological controls at different times appears to vary with variations in freshwater inflow (Yao and Hu, 2017), highlighting the importance of freshwater inflow variability in this semiarid system. While the Mission-Aransas Estuary is microtidal, the tidal fluctuations at the Aransas Ship Channel, where the autonomous sensors were located, are relatively large compared to the upper estuary and appear to exert an important control on the daily fluctuations of pH and *p*CO_2_ causing expected differences between day and night observations (based on biological activity) to reverse for portions of the year.

The co-occurrence of high *p*CO_2_, low pH, low DO, high salinity, and low temperature water is consistent with a coastal upwelling signature which is known to occur in the vicinity of Santa Monica Bay, San Francisco Bay and Tillamook Bay (Leinweber and Gruber, 2013; Raimonet and Cloern, 2017). In Tillamook Bay, this pattern was observed across consecutive years. These relationships are particularly clear in deep waters in Santa Monica Bay, while the relationships are more variable in nearshore shallower waters. The upwelling signal in West Coast water bodies was also recorded in the seasonal patterns of *p*CO_2*obs*_, *p*CO_2*thermal*_ and *p*CO_2*bio/hydro*_. This signal was particularly evident in Santa Monica Bay and San Francisco Bay, but was also observed in Tillamook Bay (Figures 8, 13; Supplementary Figures 19 – 22). Overall, the variability in *p*CO_2*thermal*_ within these West Coast water bodies is diminished compared to seasonal *p*CO_2*thermal*_ variability in GOM and East Coast water bodies due to relatively small changes in water temperature. In general, *p*CO_2*bio/hydro*_ values in Santa Monica Bay and Tillamook Bay were more closely aligned with *p*CO_2*obs*_ over the course of the year. In Santa Monica Bay (both deployments), nonthermal controls were particularly important in late winter to early spring. During this period, *p*CO_2*thermal*_ values were falling, reaching their lowest annual value well below *p*CO_2*obs*_, presumably due to cold upwelled water. Then, from the summer into the fall, *p*CO_2*thermal*_ values gradually increased while *p*CO_2*obs*_ continually decreased and tracked more closely with *p*CO_2*bio/hydro*_. Collectively, these patterns are consistent with a prominent coastal upwelling signature that is rich in CO_2_ peaking in the spring. Other studies have found that Santa Monica Bay has maximum *p*CO_2_ and minimum pH in April and May, coinciding with peak upwelling (Leinweber and Gruber, 2013; McLaughlin et al., 2018).

While the seasonal pattern of *p*CO_2*thermal*_ is similar across these West Coast sites, the relationship and timing among *p*CO_2*thermal*_, *p*CO_2*obs*_, and *p*CO_2*bio/hydro*_ are different. In Tillamook Bay, *p*CO_2*obs*_ increased from the winter into the summer, reaching its maximum in late summer–early fall before decreasing, reaching minimum values in the winter (around February). Additionally, instead of *p*CO_2*thermal*_ values reaching their minimum when *p*CO_2*obs*_ and *p*CO_2*thermal*_ were near their maximum, in Tillamook, *p*CO_2*obs*_, *p*CO_2*bio/hydro*_, and *p*CO_2*thermal*_ all reach their maximum around the same time, in late summer–early fall. Large variability in *p*CO_2_, pH, and salinity values were observed in Tillamook Bay, including the lowest and highest *p*CO_2_ values recorded across all the water bodies (all measurements considered). This may be at least in part related to strong tidal forcing in the estuary which could cause extreme mixing. Seasonal patterns were also evident, and Tillamook Bay is also known to experience periods of coastal upwelling, particularly during spring and summer months (Colbert and McManus, 2003). Upwelling of cold, saline rich waters during the summer have also been observed in the nearby Salish Sea (Cai et al., 2021). The upwelling signal in Tillamook Bay was attenuated as compared to Santa Monica Bay and San Francisco Bay. At least some of this attenuation was likely due to the location of the sensor packages in the estuary (close to shore/shallower in Tillamook vs. further offshore, deeper water in Santa Monica Bay). Tillamook Bay has high freshwater inflow relative to estuarine volume and as a result, the upwelling signal is modified by riverine inputs, *in situ* biological uptake and regeneration processes within the bay (Colbert and McManus, 2003). This is supported by observations of diminished freshwater flow in the summer and increased freshwater flow in the winter driven by seasonal variations in precipitation. As such, coastal upwelling along with freshwater inputs may be playing particularly important roles in controlling *p*CO_2_ variability during these months.

In Casco Bay, peak *p*CO_2*thermal*_ values occurred in August and then continuously decreased until January. This pattern was consistent across consecutive years of data collection. *p*CO_2*obs*_ values continued to increase over this same period as *p*CO_2*thermal*_ was decreasing, reaching their maximum a couple of months later, in October. *p*CO_2*bio/hydro*_ values continued to increase from August to October, similar to the pattern of *p*CO_2*obs*_. Collectively, these patterns indicate a significant nonthermal control on *p*CO_2*obs*_ characterized by enhanced biological activity in the spring resulting in enhanced removal of CO_2_ from the water column and then the return of CO_2_ to the water column in the late summer and fall. Only a few months of data are available for Barnegat Bay, therefore seasonal patterns among *p*CO_2_ and the various transformations were difficult to distinguish.

Recent work has shown that observed amplitudes of seasonal pH and *p*CO_2_ cycles increase moving landward from coastal ocean to nearshore and estuarine monitoring stations (Fassbender et al., 2018). The dynamic seasonal cycles observed in the estuarine pH and *p*CO_2_ monitoring data discussed here are consistent with this pattern, as well as with other published estuarine datasets (e.g., Hofmann et al., 2011; Wallace et al., 2014; Joesoef et al., 2015; Fairchild and Hales, 2021; Jiang et al., 2021; Torres et al., 2021). This highlights the relatively extreme carbonate chemistry experienced in estuaries compared to open ocean environments, and the continuing need to consider how ongoing coastal acidification will modify the seasonality of carbonate chemistry cycles (Fassbender et al., 2018). This work will be critical to inform our understanding of how coastal acidification manifests in nearshore and estuarine habitats, including how changing seasonality may control the timing of exceedance of water quality and biological thresholds (Pacella et al., 2018; Cai et al., 2021).

### Diel Variability in *p*CO_2_ and pH

Shorter-term, diel trends observed in the data are superimposed on the longer-term trends (Figure 13). These trends collectively reflect the influence of weather, tides, and biological activity (e.g., productivity, respiration), drivers that have been documented in other coastal systems (e.g., Yates et al., 2007; Dai et al., 2009; Pacella et al., 2018; Shen et al., 2019). For example, DO and *p*CO_2_ relationships can be indicative of photosynthesis and respiration processes and large ranges of pH and DO indicate more photosynthetic activity and potentially nutrient enrichment. When deviations in DO-pH relationships are observed, other factors may be controlling dynamics, such as freshwater input and upwelling. The influence of tidal fluctuations on seawater chemistry is evident in the regular pattern of salinity variations. For example, in Casco Bay and Tampa Bay large diel ranges in salinity (~4 Practical Salinity Units [PSU]) are observed which are likely related to tidal cycles. As expected, in the deeper Santa Monica Bay deployment, the pattern is irregular, and the salinity signal was muted with daily variability on the order of ~0.2 PSU; this is because this is an open coast site. Daily patterns of DO and *p*CO_2_ were also evident wherein increases in *p*CO_2_ corresponded to decreases in DO concentrations, and vice versa. This could be related to the relative influence of productivity and respiration throughout the day. The timing of these day/night patterns (intervals) was quite regular in the shallower Casco Bay, Tampa Bay, and Santa Monica Bay (15 m) deployments but less so in the deeper Santa Monica Bay (60 m) deployment. More detailed analyses outside the scope of this study are needed to separate the day-night signal from the diurnal and semi-diurnal tidal signal.

## CONCLUSION

The United States EPA’s National Estuary Programs (NEP) along with their partnering institutions and agencies are expanding the use of autonomous monitoring *p*CO_2_ and pH sensors to evaluate carbonate chemistry in estuarine environments. Analysis of year to multi-year data from seven NEPs indicate that the sensors effectively captured key parameter measurements enabling an initial characterization of daily to seasonal trends in carbonate chemistry across a range of environmental settings. This study documented extreme variability in coastal acidification data within, and across, a range of coastal systems. Interestuary annual *p*CO_2_ and pH variability up to 1,263 μatm (Tillamook Bay) and 1.16 pH units (Casco Bay) respectively were observed. Across all water bodies, temperature and *p*CO_2_ were, in general, lower in cooler, winter months and higher in warmer, summer months; *p*CO_2_ and pH values were also more variable in warmer months. As expected, clear coastal upwelling signals were observed in the monitoring data from Santa Monica Bay, San Francisco Bay, and Tillamook Bay. Evidence of both thermal and nonthermal controls on *p*CO_2_ were observed in these systems (i.e., seasonal net community productivity, tidal fluctuations, and freshwater inflows). Evidence of tidal control and day-night changes (e.g., daily temperature variability and diurnal variability in the relative contribution of photosynthesis and respiration) were observed. The relative influence of these drivers should be evaluated as more data becomes available.

Acidification is occurring globally but is a particular concern in coastal zones where local amplifiers such as upwelling, eutrophication, and freshwater inflows combined with human activities exist. The buffering capacity in coastal systems varies as a result of multiple environmental factors. Continued high-resolution monitoring will be critical for understanding how these coastal systems are changing and for identifying and quantifying the factors that contribute to coastal acidification, classifying the susceptibility of these water bodies to the impacts of acidification, and determining which mitigation and adaptive management strategies could be most impactful.

We encourage the continued collection of long-term coastal acidification monitoring data in the system discussed here and expansion of monitoring to other coastal systems. As additional data become more available and patterns of water quality parameter associations become clearer over time, management bodies may use the information to inform management decisions for their estuaries. Recommended next steps could include a more in-depth analysis of the various drivers of acidification in each of these coastal systems through integration of high-resolution monitoring data with other types of data, such as precipitation data, estimates of nutrient inputs, freshwater flows, and upwelling models to inform acidification drivers.

## Supplementary Material

Supplement1

## Figures and Tables

**TABLE 1 |:** Autonomous sensors used to monitor water chemistry in seven of United States Environmental Protection Agency’s (EPA’s) National Estuary Programs (NEPs).

Water Body	Autonomous Deployment Instruments			
	CTD^a^	*p*CO_2_	pH	DO
Santa Monica Bay	Sea-Bird SeapHOx	Sunburst SAMI-CO_2_	Sea-Bird SeapHOx	Sea-Bird SeapHOx
San Francisco Bay	Sea-Bird SeapHOx	MAPCO_2_	Sea-Bird SeapHOx and Sea-Bird SeaFET	Sea-Bird SeapHOx
Tillamook Bay	Sea-Bird SeapHOx and YSI	SunBurst SAMI-CO_2_	Sea-Bird SeapHOx and Sea-Bird SeaFET	Sea-Bird SeapHOx and YSI
Barnegat Bay	YSI	Pro-Oceanus CO_2_-Pro CV	Sea-Bird SeaFET	YSI
Casco Bay	Sea-Bird CTD	SunBurst SAMI-CO_2_	Sea-Bird SeaFET	Aanderaa Oxygen Optode
Mission-Aransas Estuary	YSI	SunBurst SAMI-CO_2_	Sea-Bird SeaFET	YSI
Tampa Bay	Sea-Bird SeapHOx	Pro-Oceanus CO_2_-Pro CV	Sea-Bird SeapHOx	Sea-Bird SeapHOx

*p*CO_2_, partial pressure of carbon dioxide; DO, dissolved oxygen.

a A variety of electronic instrument packages were used by the NEPs to measure conductivity, temperature, and depth (CTD).

**TABLE 2 |:** Sensor Specifications.

Instrument	Parameter	Accuracy	Precision	Resolution	Range
SunBurst SAMI-CO_2_	*p*CO_2_	+/− 3 μatm	± 0.5–1 μatm		150–700^a^
Pro-Oceanus CO_2_-Pro CV	*p*CO_2_	± 0.5% of meas. val.	0.01 ppm		0–10,000
MAPCO_2_^b^	*p*CO_2_	< 2 μatm	0.7 ppm		0–800
Sea-Bird SeapHOx	pH	± 0.05		±0.004	6.5–9
	DO	± 0.1 mg L^−1^		0.2 μmol kg^−1^	120% of surf. sat.
	Temp	± 0.002°C^c^ ± 0.01°C^d^		0.0001°C	−5 to 45°C
Satlantic SeaFET	pH	± 0.05	± 0.004		6.5–9
Aanderaa Oxygen Optode	DO	< 8 μM		<0.1 μM	0–1,000 μM

a instrument can be calibrated for extended ranges.

b LiCOr LI-820 CO_2_ gas analyzer (Sutton et al., 2014).

c Temperature range: −5 to 35°C.

d Temperature range: 35 to 45°C.

## Data Availability

The datasets presented in this study can be found in online repositories. The original data described in this manuscript can be accessed at NOAA’s National Centers for Environmental Information (NCEI) at https://doi.org/10.25921/xg33-1n83. Data generated during this study for Tampa Bay are available as a USGS data release (Yates et al., 2019): https://coastal.er.usgs.gov/data-release/doi-P9BAFC7L/.

## Footnotes

1
https://www.epa.gov/nep

2
https://www.epa.gov/quality/quality-assurance-project-plan-development-tool

3
https://doi.org/10.25921/xg33-1n83
