# Supplementary material for: Integrating High-Resolution Coastal Acidification Monitoring Data Across Seven United States Estuaries
Source: Front Mar Sci. Author manuscript; Available in PMC 2022 Aug 19. (PMC9179233; doi:10.3389/fmars.2021.679913)
Supplement: Supplement1 [file NIHMS1790793-supplement-Supplement1.docx]

Supplementary Material

**Disclaimer.** Any use of trade, firm, or product names is for descriptive purposes only and does not imply endorsement by the U.S. Government.

# Supplementary Figures


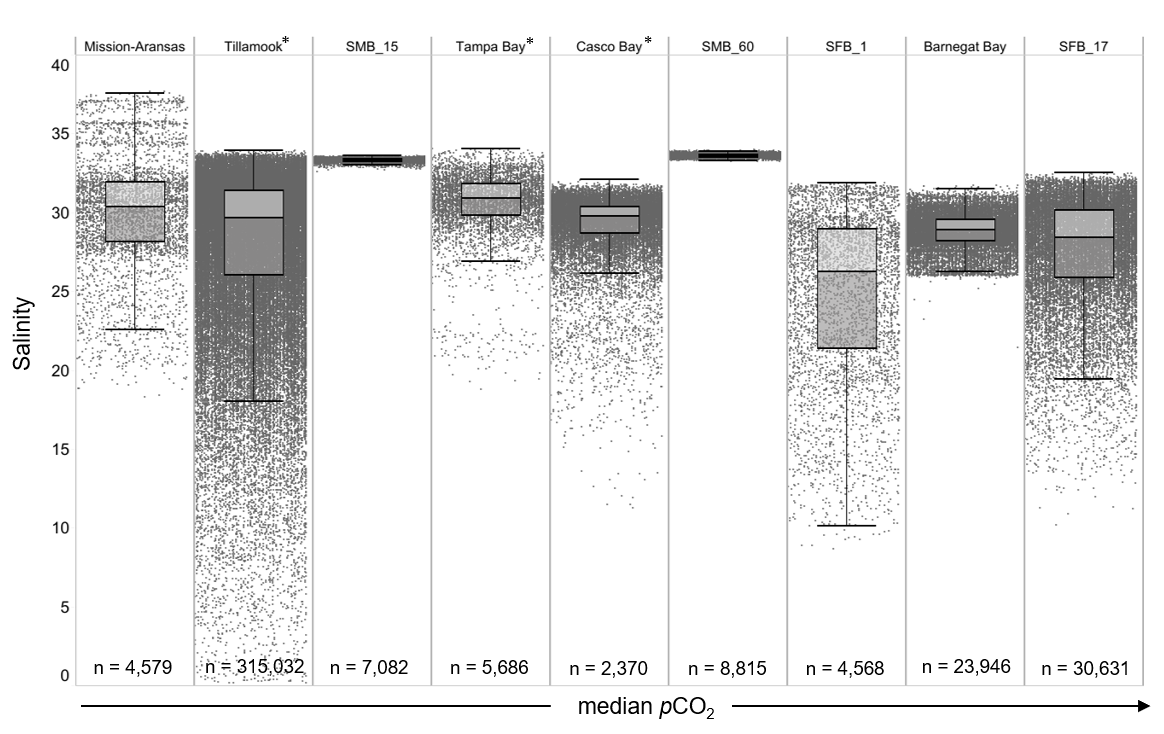


**Supplementary Figure 1.** Box plots and underlying data showing the distribution of salinity data across the seven water bodies. The water bodies are arranged from lowest median partial pressure of carbon dioxide (*p*CO_2_) (Mission-Aransas Estuary) to highest median *p*CO_2_ (Barnegat Bay) as calculated from all available data. Whiskers extend to data within 1.5 times the interquartile range; * = multi-year records. SMB. SMB_15 = Santa Monica Bay (15 m deployment); SMB_60 = Santa Monica Bay (60 m deployment); SFB_1 = San Francisco Bay (1 m deployment); SFB_17 = San Francisco Bay (17 m deployment).


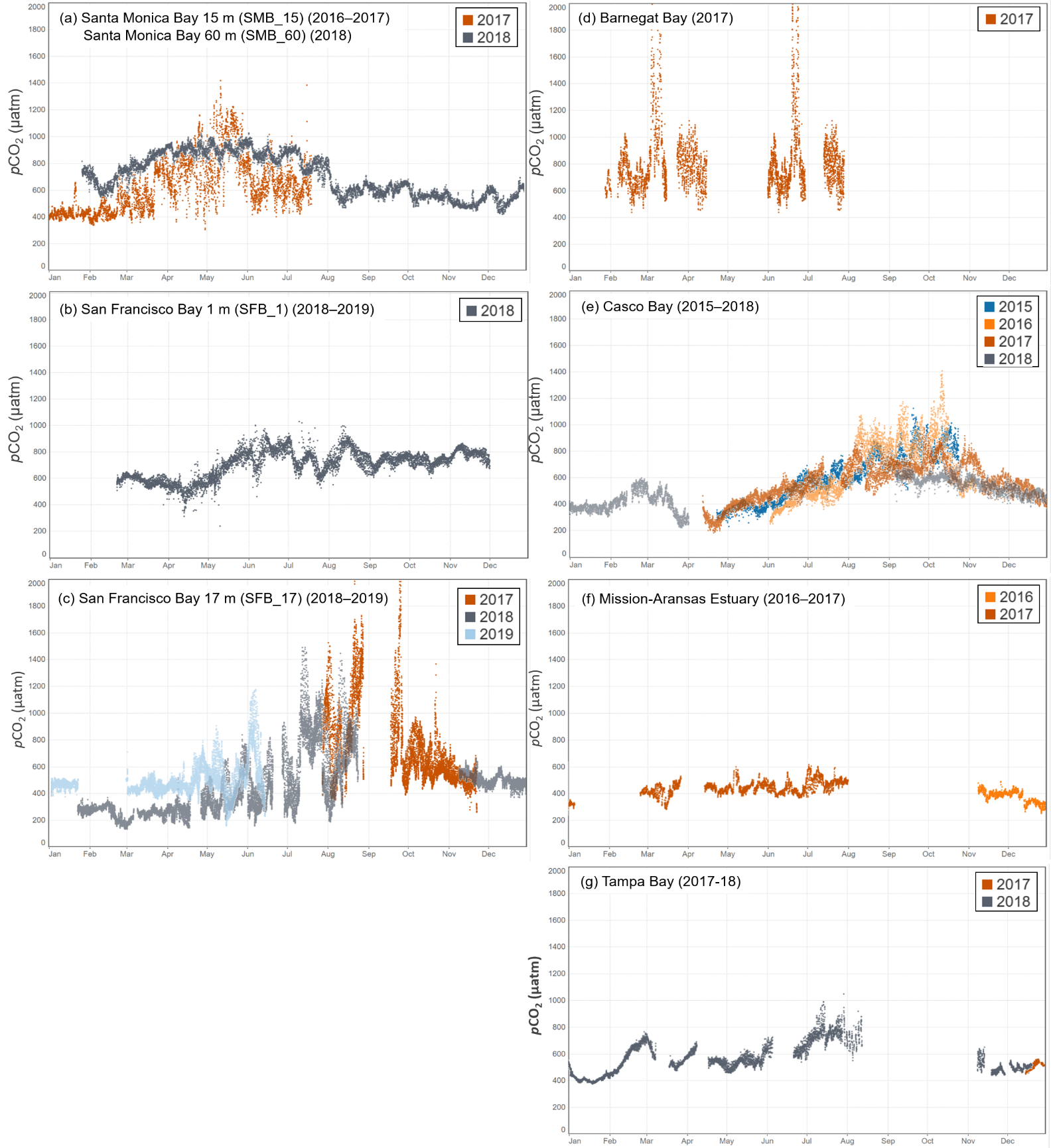


(c) Tillamook Bay (2017–2019)

**Supplementary Figure 2.** Time series plots of observed partial pressure of carbon dioxide (*p*CO_2_) in each of seven National Estuary Program water bodies keyed out by year: (a) Santa Monica Bay 15 m; Santa Monica Bay 60 m; (b) San Francisco Bay 1 m; (c) Tillamook Bay; (d) Barnegat Bay; (e) Casco Bay; (f) Mission-Aransas Estuary; (g) Tampa Bay.


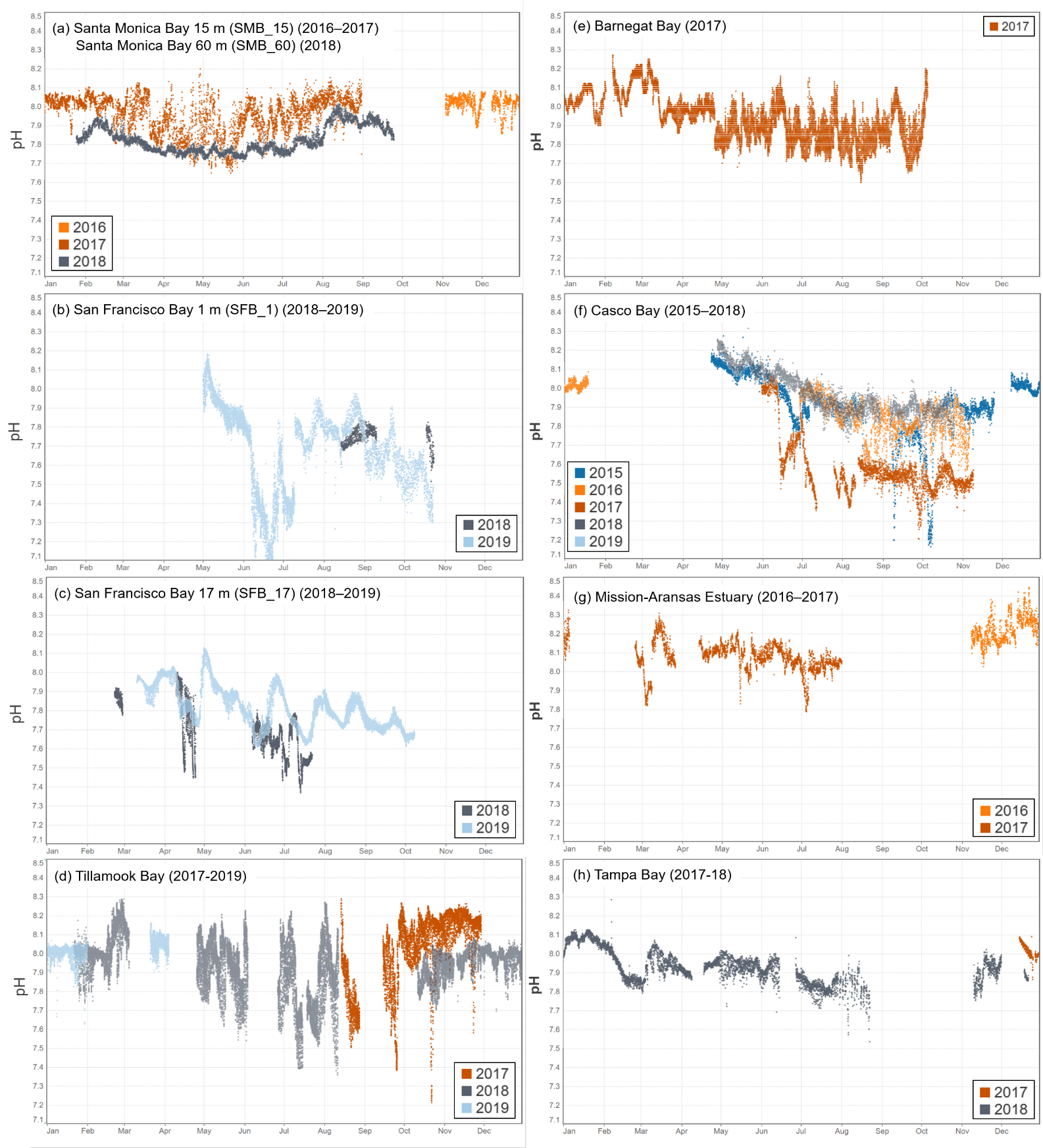
**Supplementary Figure 3.** Time series plots of observed pH within each water body keyed out by year. (a) Santa Monica Bay 15 m; Santa Monica Bay 60 m; (b) San Francisco Bay 1 m; (c) San Francisco Bay 17 m; (d) Tillamook Bay; (e) Barnegat Bay; (f) Casco Bay; (f) Mission-Aransas Estuary; (h) Tampa Bay.


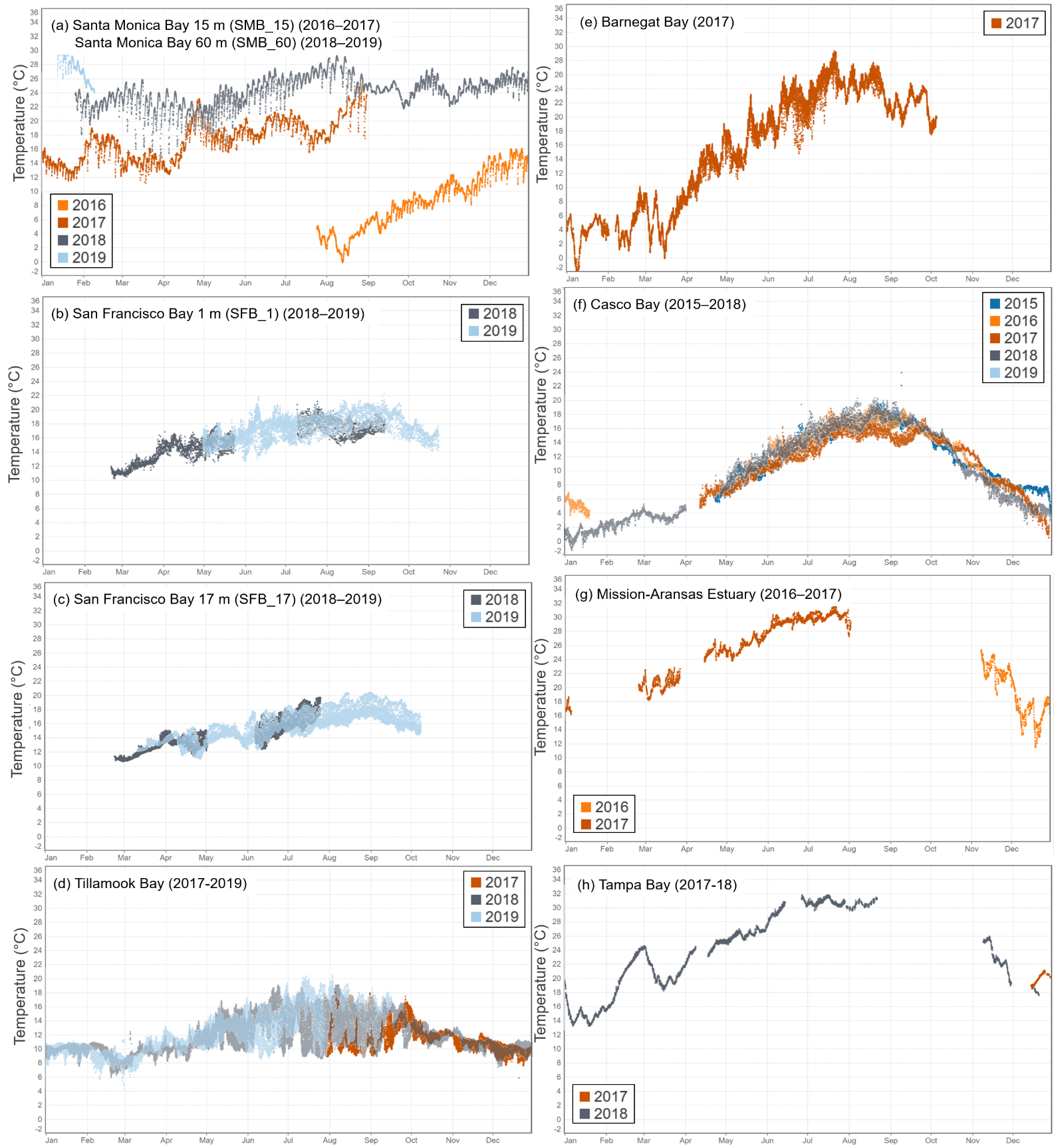


**Supplementary Figure 4.** Time series plots of observed temperature within each water body keyed out by year. (a) Santa Monica Bay 15 m; Santa Monica Bay 60 m; (b) San Francisco Bay 1 m; (c) San Francisco Bay 17 m; (d) Tillamook Bay; (e) Barnegat Bay; (f) Casco Bay; (f) Mission-Aransas Estuary; (h) Tampa Bay.


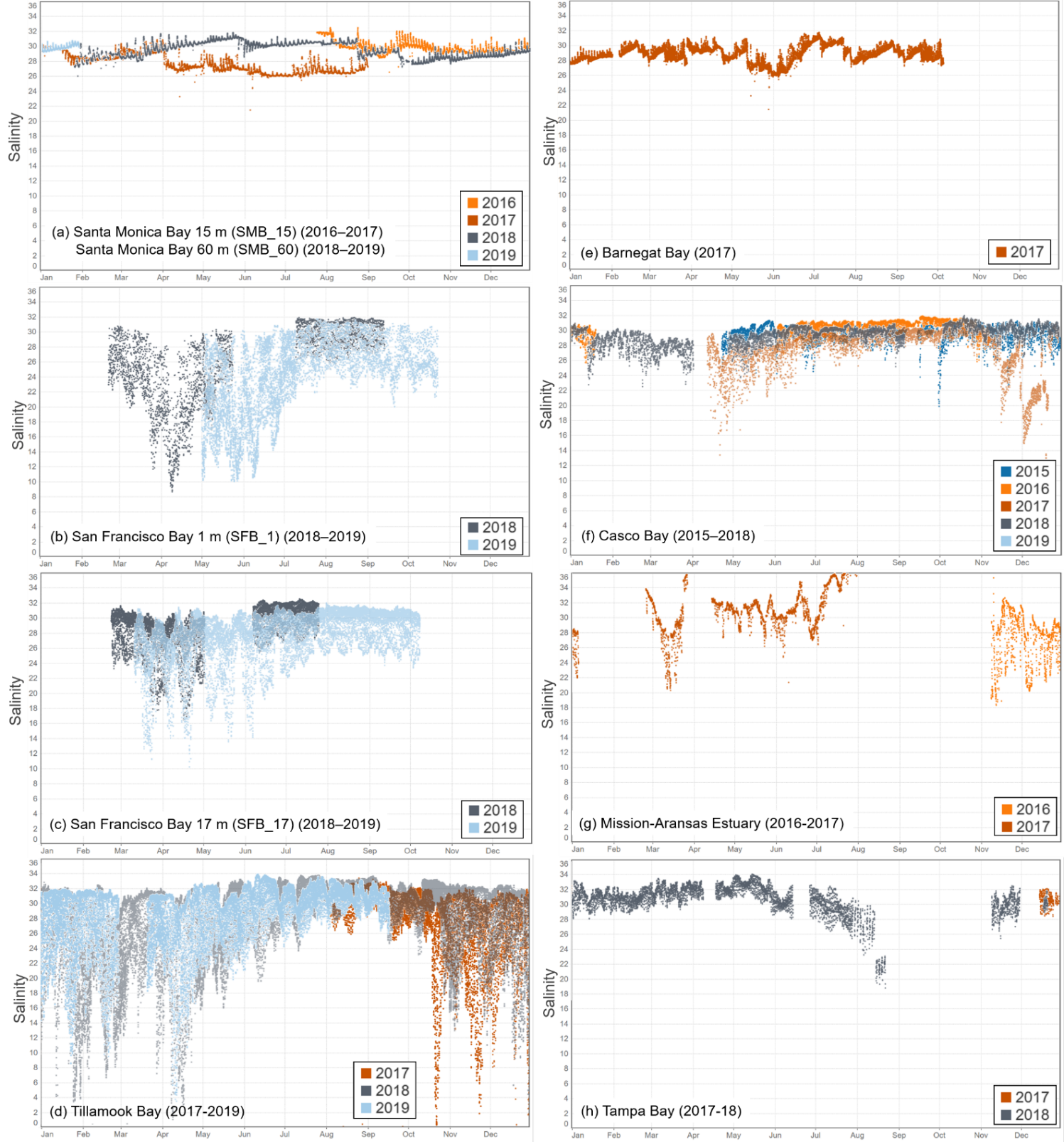


**Supplementary Figure 5.** Time series plots of observed salinity within each water body keyed out by year. (a) Santa Monica Bay 15 m; Santa Monica Bay 60 m; (b) San Francisco Bay 1 m; (c) San Francisco Bay 17 m; (d) Tillamook Bay; (e) Barnegat Bay; (f) Casco Bay; (f) Mission-Aransas Estuary; (h) Tampa Bay.


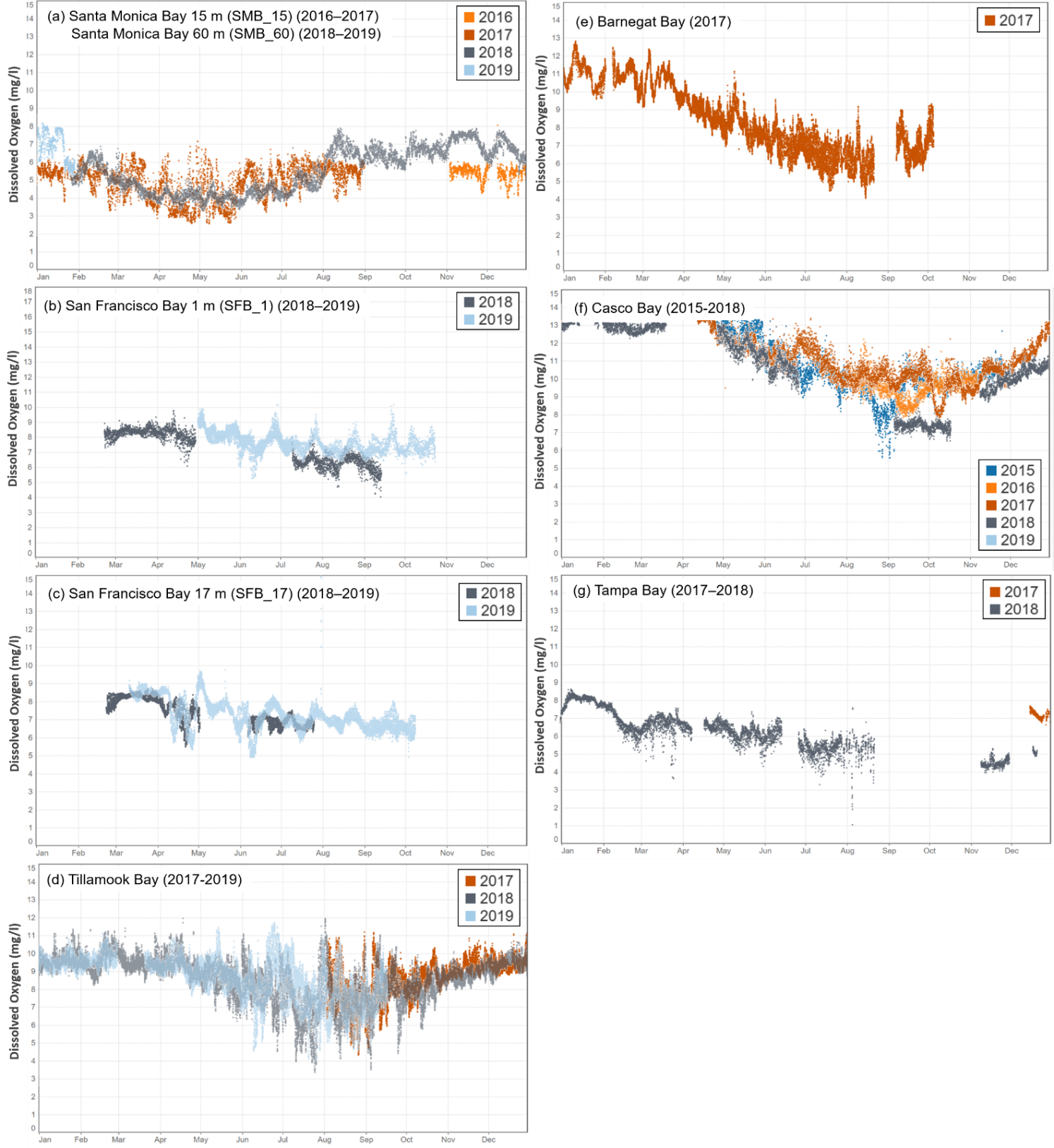


**Supplementary Figure 6.** Time series plots of observed dissolved oxygen (DO) within each water body keyed out by year. (a) Santa Monica Bay 15 m; Santa Monica Bay 60 m; (b) San Francisco Bay 1 m; (c) San Francisco Bay 17 m; (d) Tillamook Bay; (e) Barnegat Bay; (f) Casco Bay; (f) Mission-Aransas Estuary; (h) Tampa Bay.


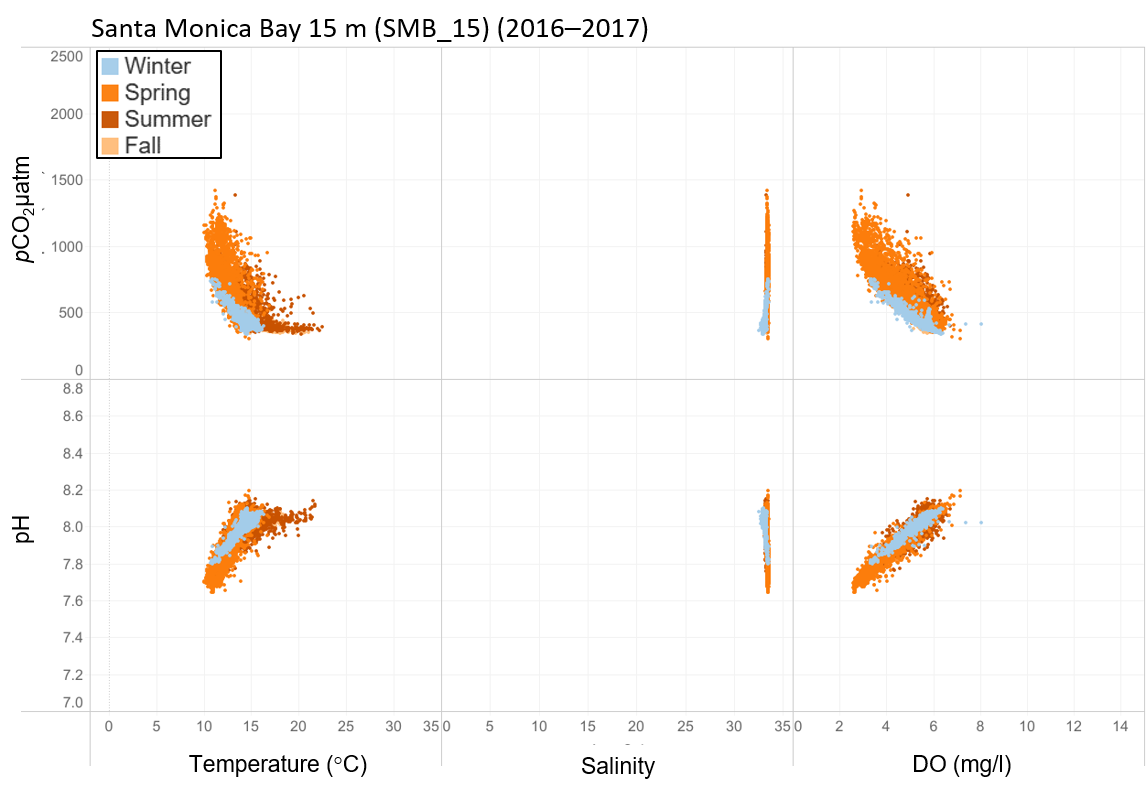


**Supplementary Figure 7.** Cross-plots highlighting relationships among temperature/salinity/DO and pH/*p*CO_2_ in Santa Monica bay data collected at ~15 m below the surface. Data colored by season.


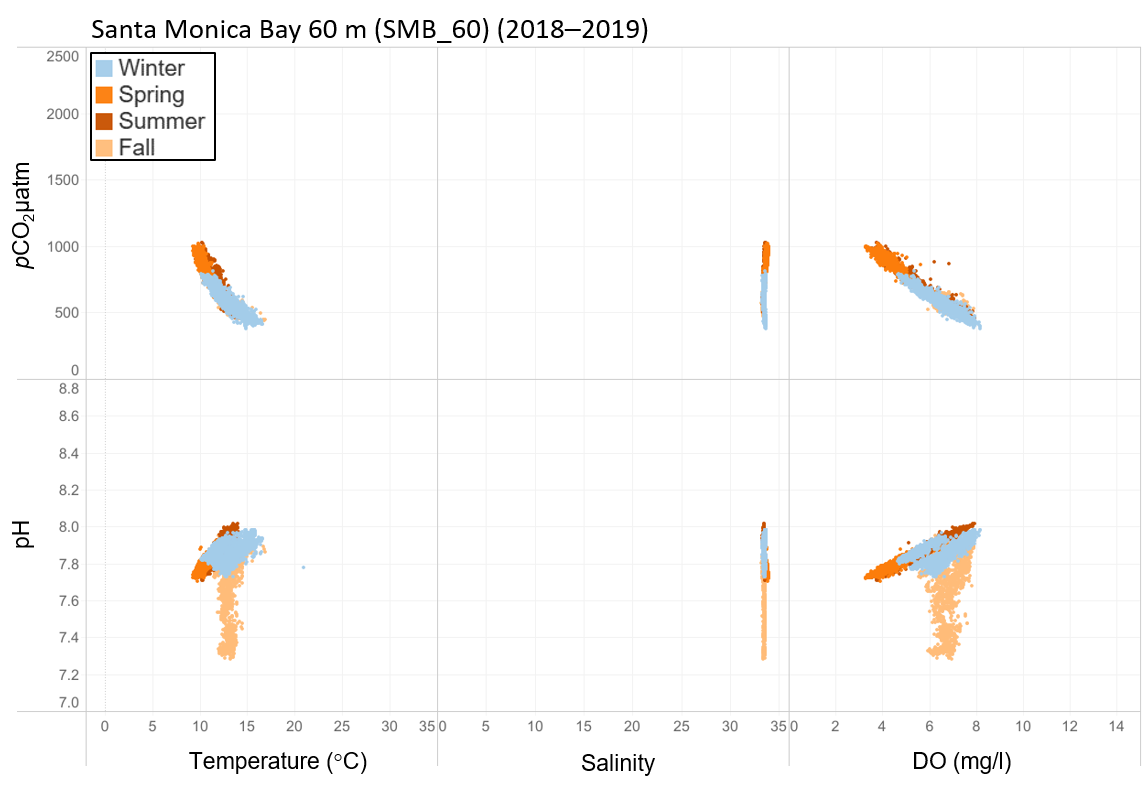


**Supplementary Figure 8.** Cross-plots highlighting relationships among temperature/salinity/DO and pH/*p*CO_2_ in Santa Monica Bay collected at ~60 m below the surface. Data colored by season.


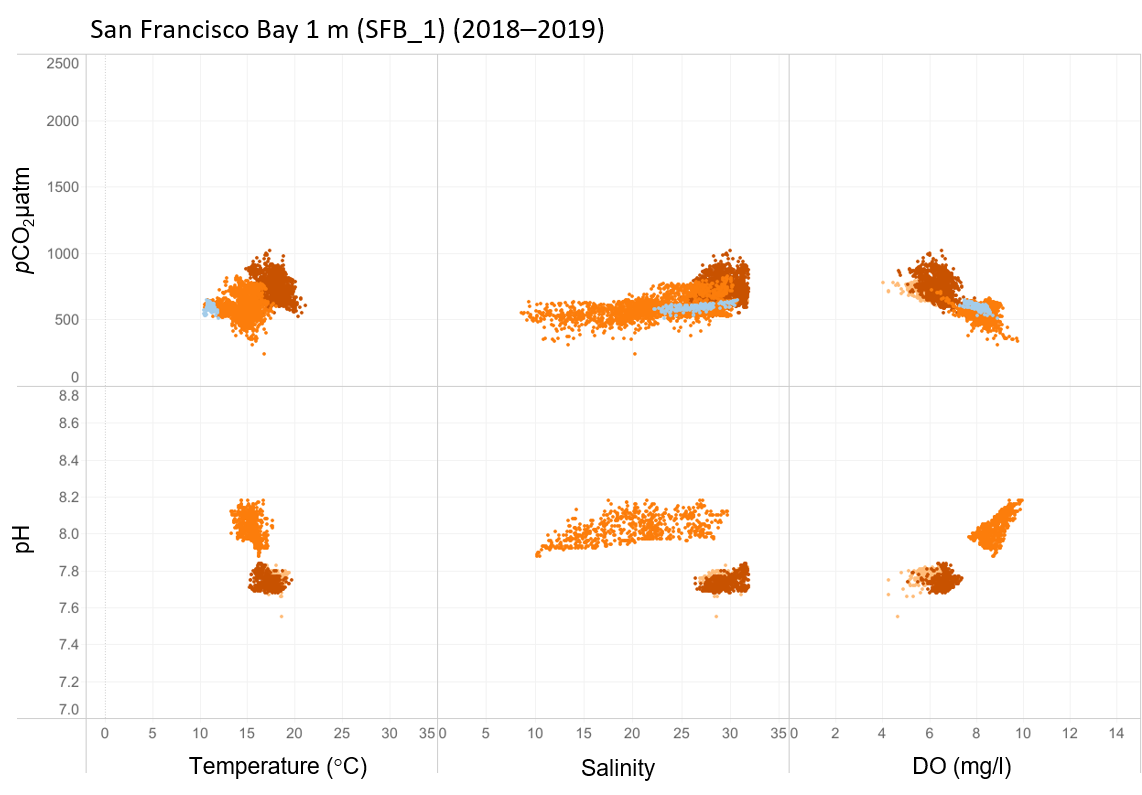


**Supplementary Figure 9.** Cross-plots highlighting relationships among temperature/salinity/DO and pH/*p*CO_2_ in San Francisco Bay collected at ~1 m below the surface. Data colored by season.


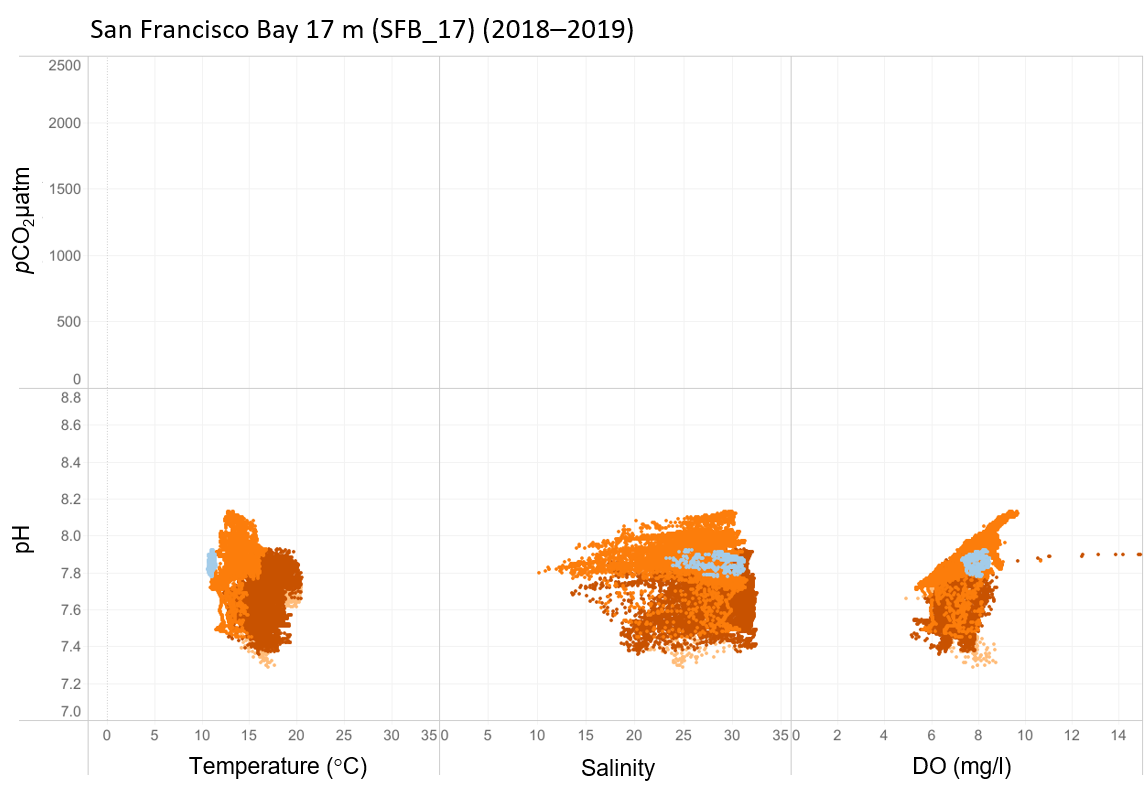


**Supplementary Figure 10.** Cross-plots highlighting relationships among temperature/salinity/DO and pH/*p*CO_2_ in San Francisco Bay collected at ~17 m below the surface. Data colored by season.


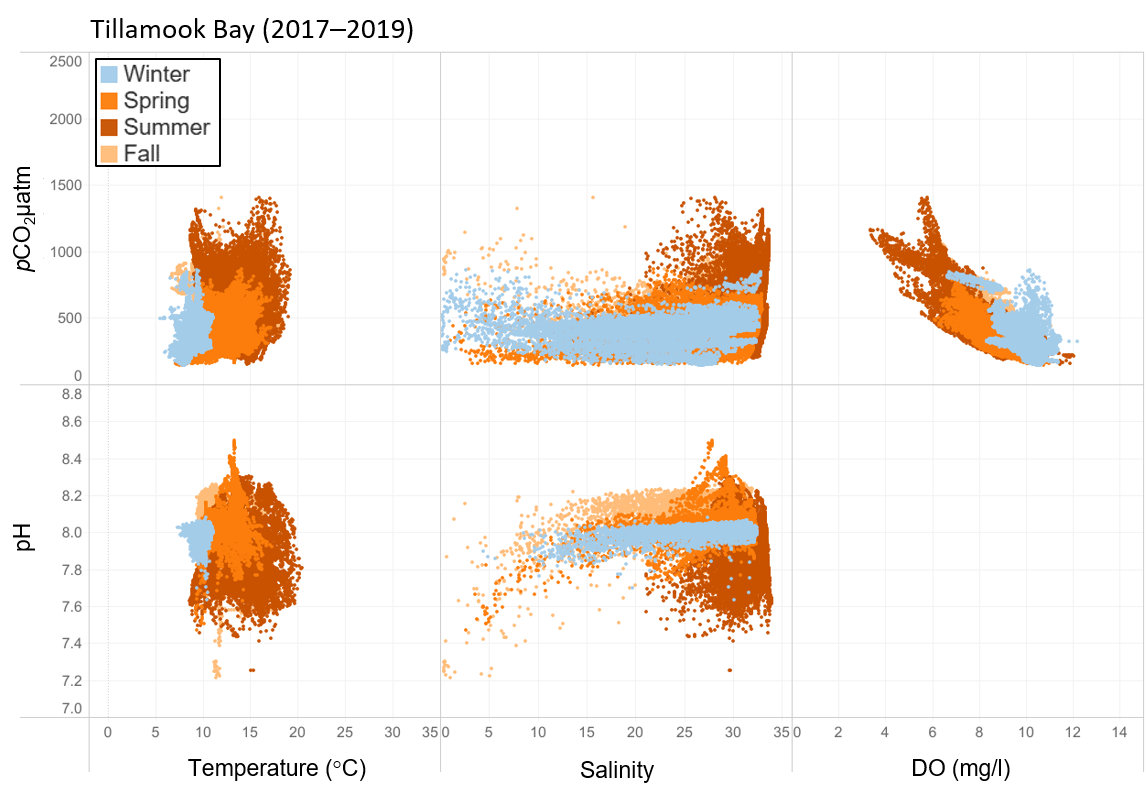


**Supplementary Figure 11.** Cross-plots highlighting relationships among temperature/salinity/DO and pH/*p*CO_2_ in in Tillamook Bay. Data colored by season.


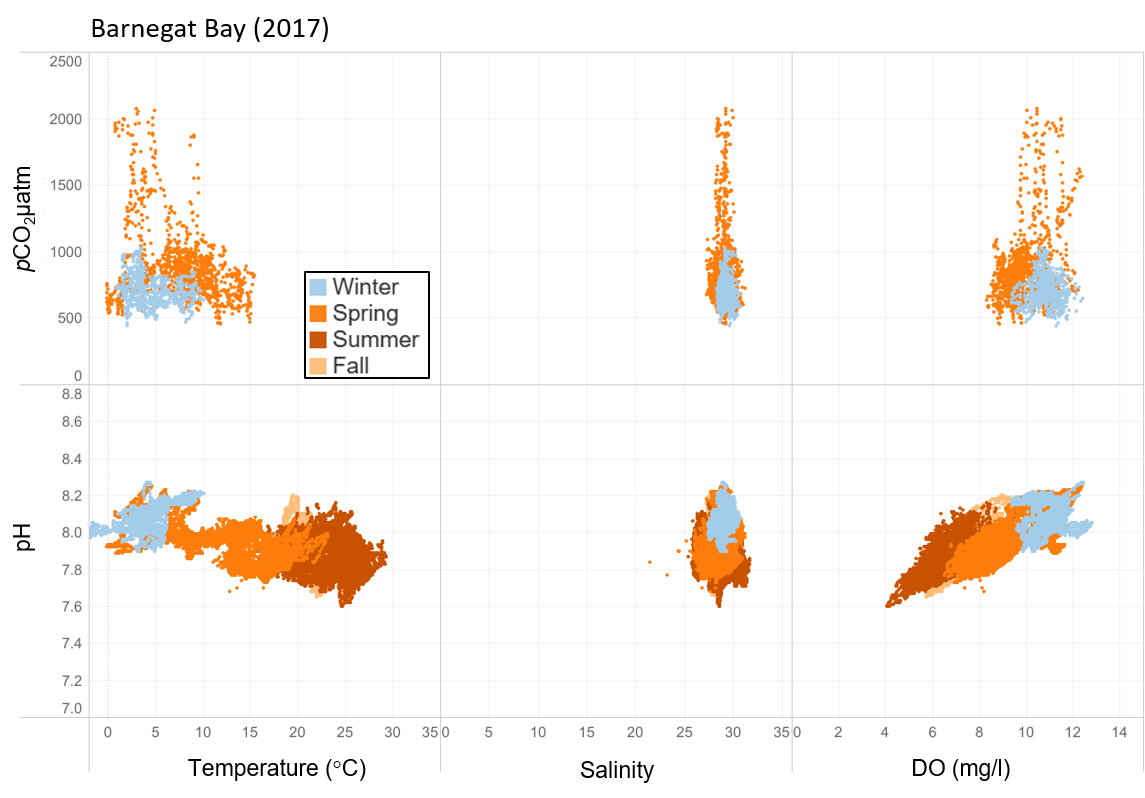


**Supplementary Figure 12.** Cross-plots highlighting relationships among temperature/salinity/DO and pH/*p*CO_2_ in Barnegat Bay. Data colored by season.


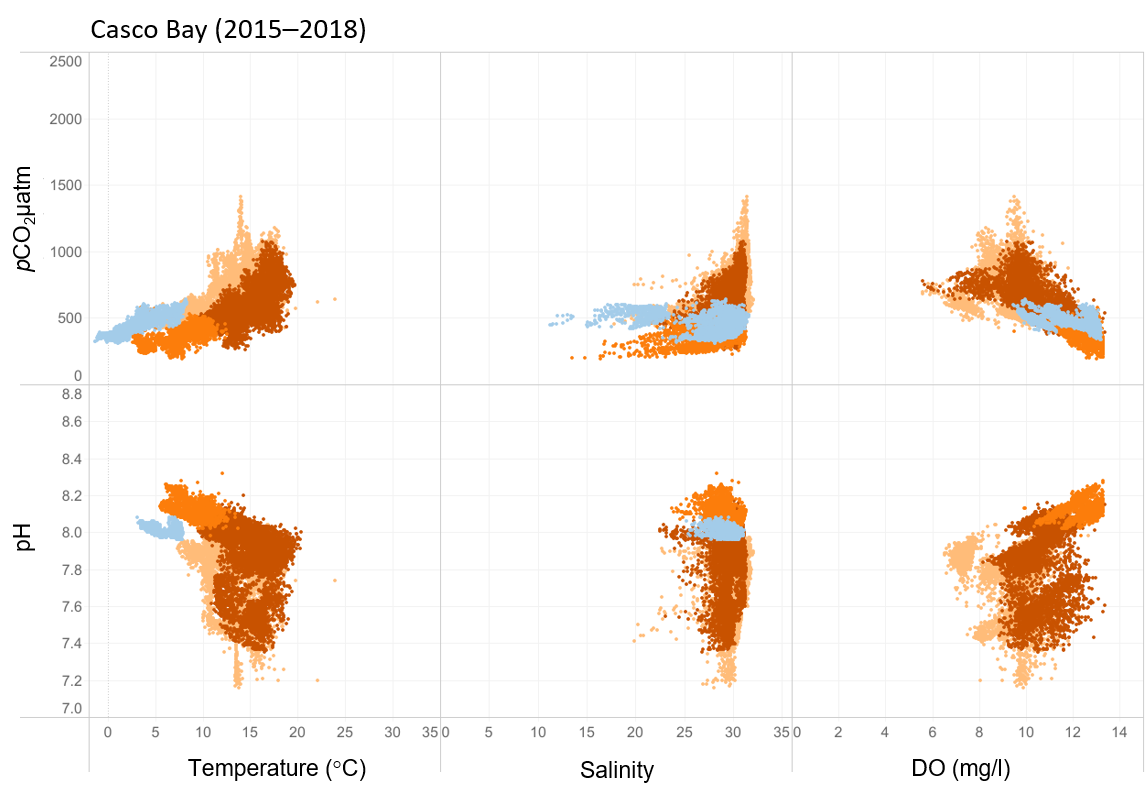


**Supplementary Figure 13.** Cross-plots highlighting relationships among temperature/salinity/DO and pH/*p*CO_2_ in Casco Bay. Data colored by season.


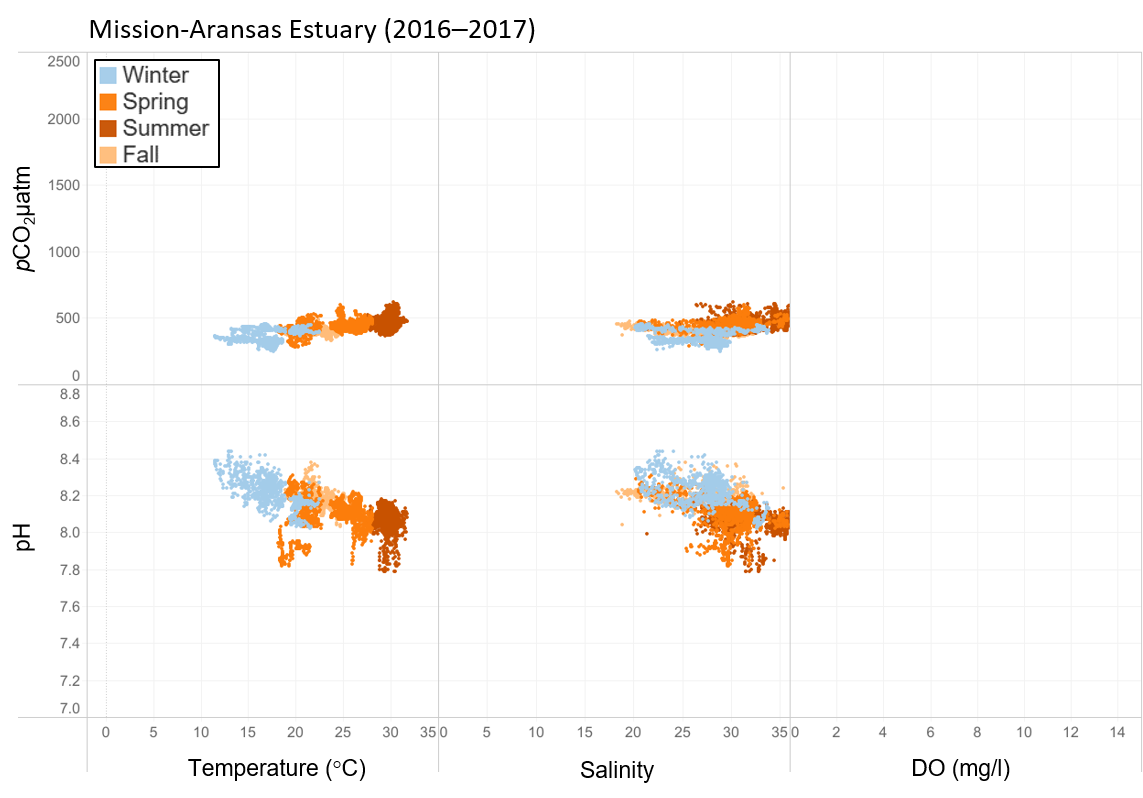


**Supplementary Figure 14.** Cross-plots highlighting relationships among temperature/salinity/DO and pH/*p*CO_2_ in Mission – Aransas Bay. Data colored by season.


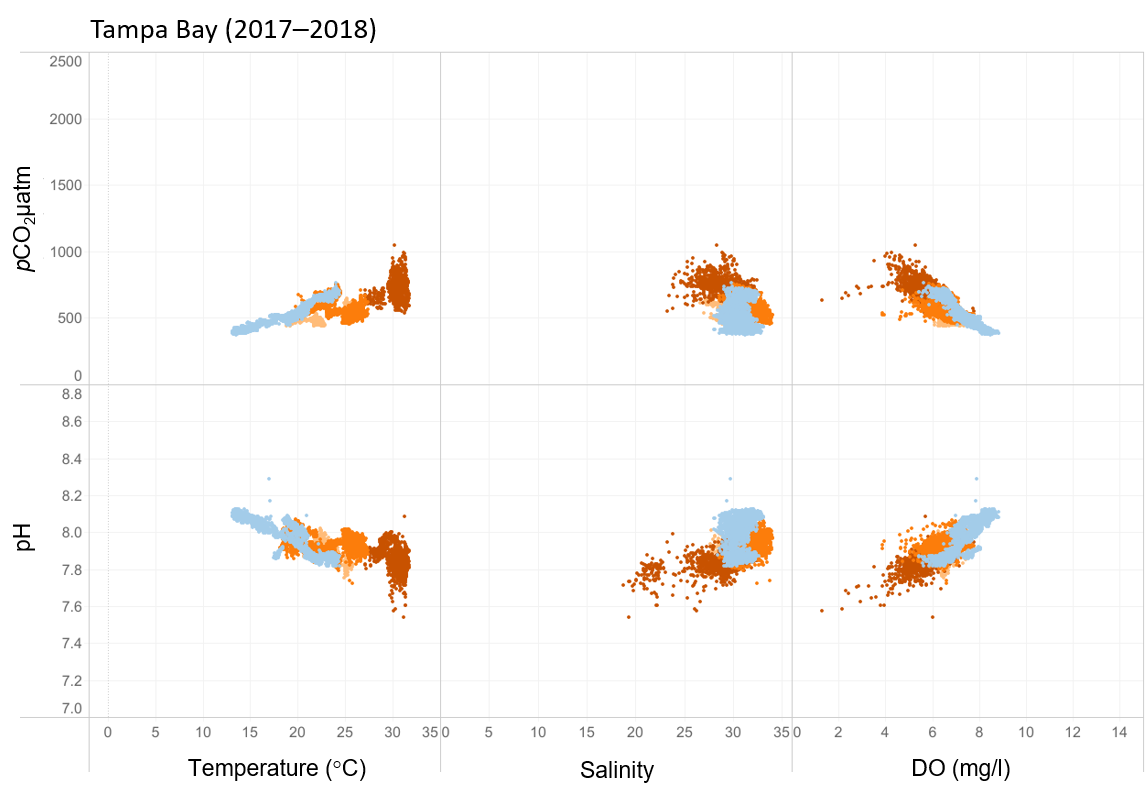


**Supplementary Figure 15.** Cross-plots highlighting relationships among temperature/salinity/DO and pH/*p*CO_2_ in Tampa Bay. Data colored by season.


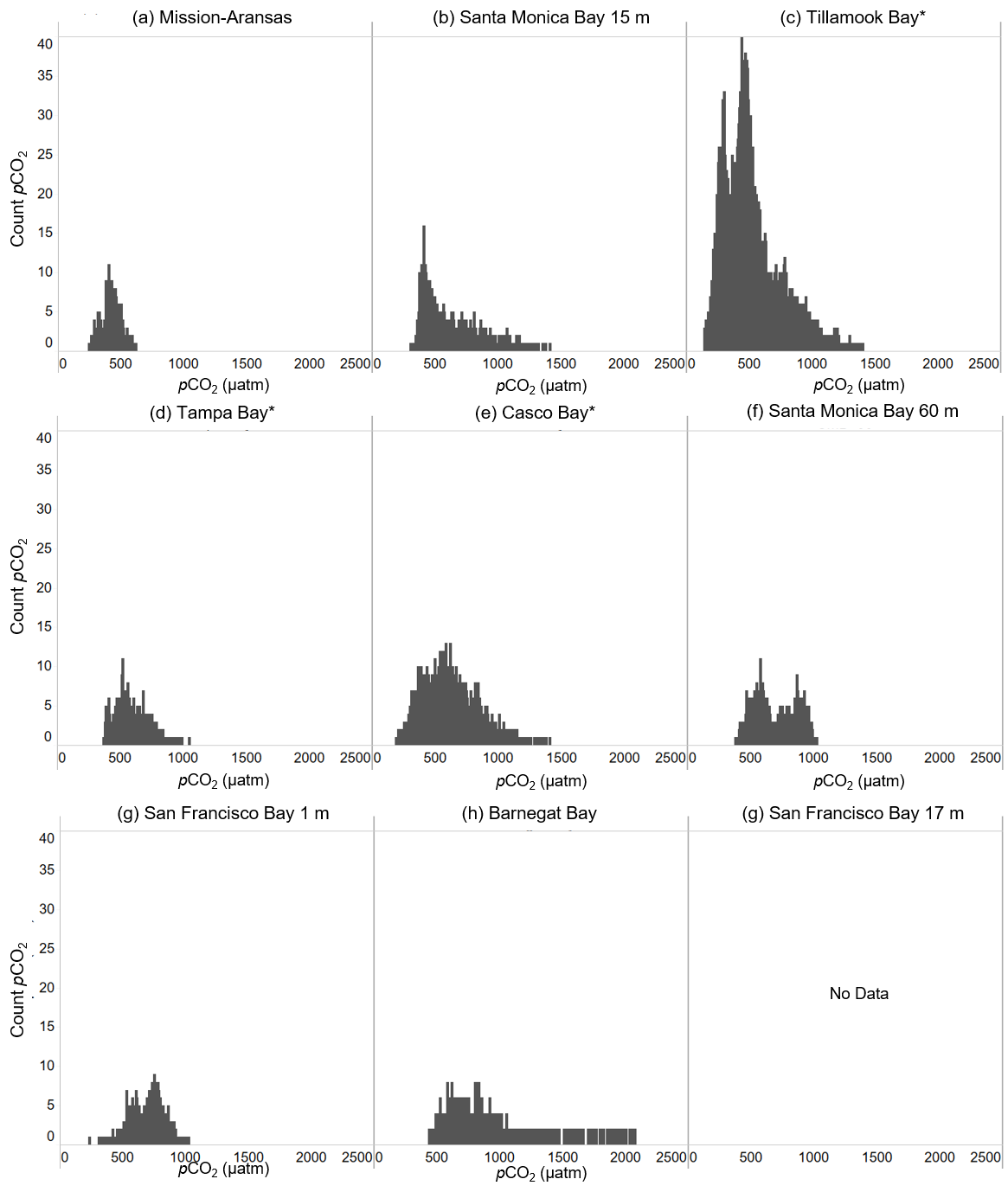


**Supplementary Figure 16.** Frequency distribution of measured *p*CO_2_. * = multi-year records. Water bodies arranged in order of increasing median *p*CO_2_.


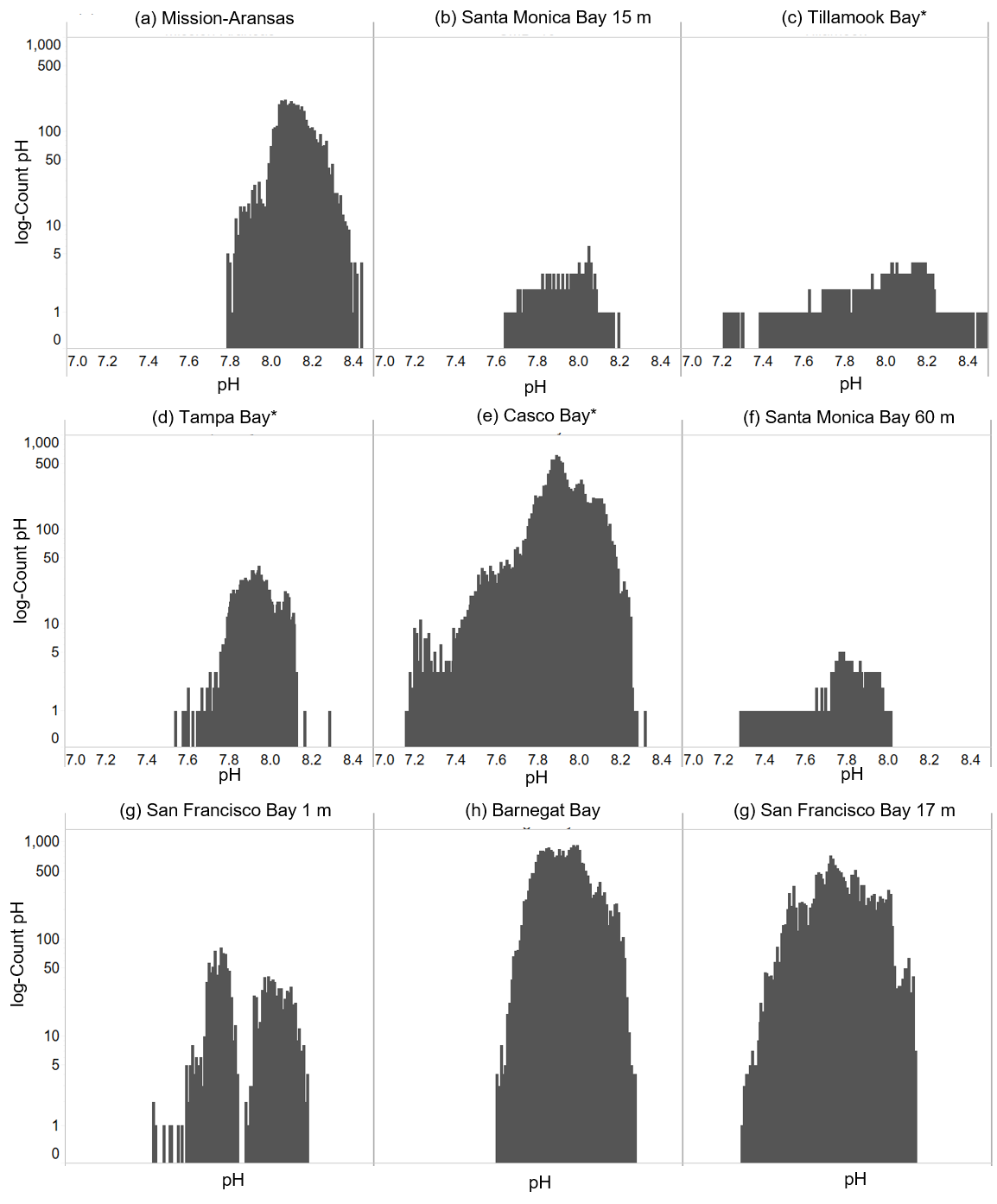


**Supplementary Figure 17.** Frequency distribution of measured pH. * = multi-year records. Water bodies arranged in order of increasing median *p*CO_2_.


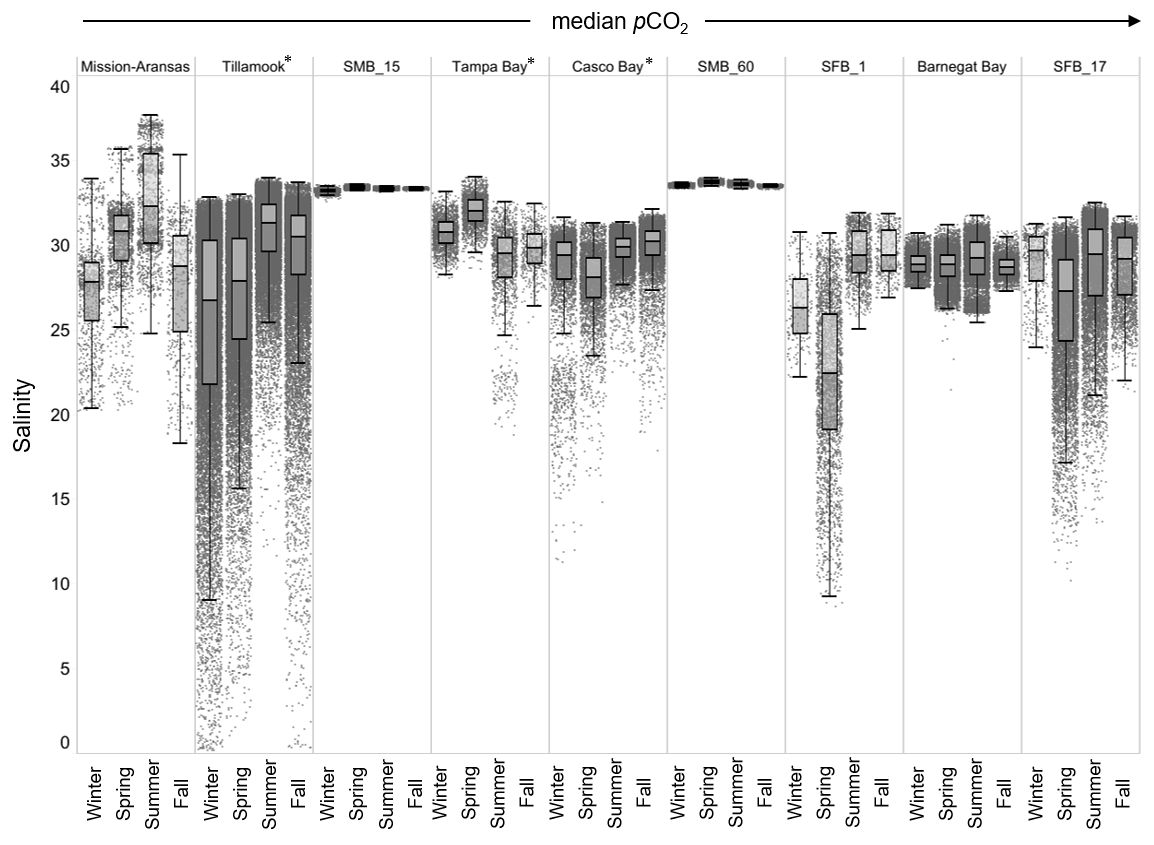


**Supplementary Figure 18.** Box plots and underlying data showing the seasonal distribution of salinity data across the seven water bodies. The water bodies are arranged from lowest median partial pressure of carbon dioxide (*p*CO_2_) to highest median *p*CO_2_. Whiskers extend to data within 1.5 times the interquartile range (IQR). Northern hemisphere meteorological season (winter – December, January, February; spring – March, April, May; summer – June, July, August; fall – September, October, November. * = multi-year records. SMB_15 =


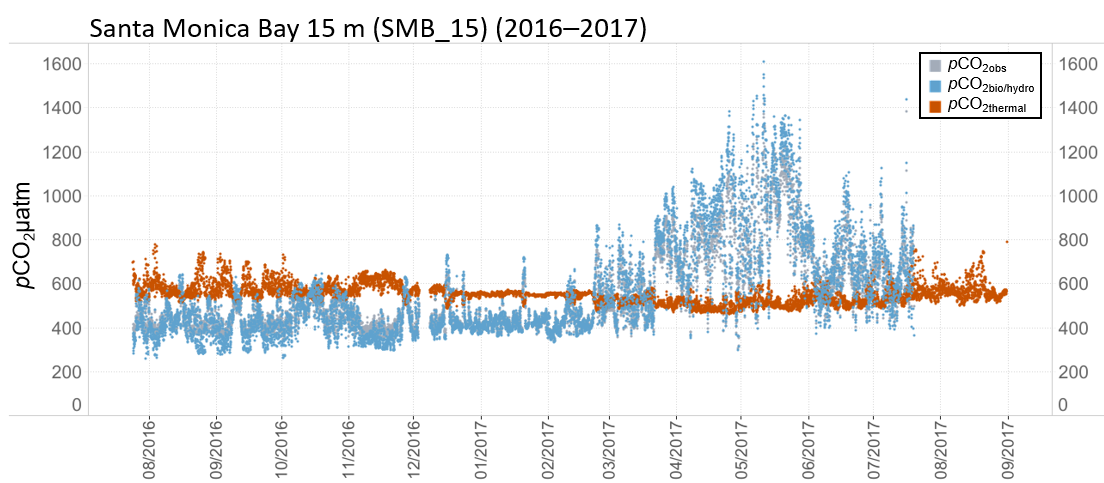


**Supplementary Figure 19.** Observed *p*CO_2_ (*p*CO_2obs_ – gray symbols), temperature-normalized (*p*CO_2bio/hydro_ – blue symbols) and *p*CO_2_-normalized (*p*CO_2thermal_ – red symbols) data collected in Santa Monica Bay at a depth of ~ 15 m.

**
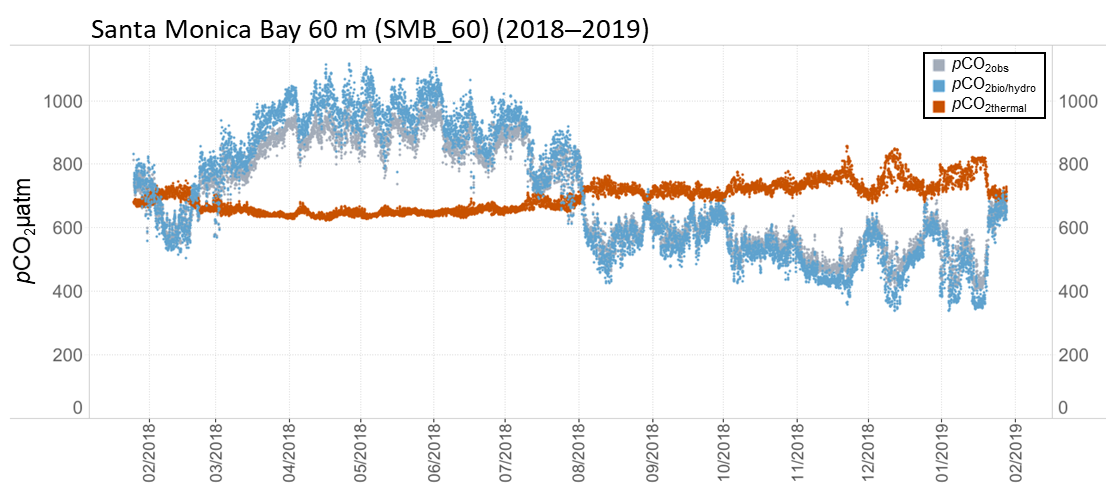
**

**Supplementary Figure 20.** Observed *p*CO_2_ (*p*CO_2obs_ – gray symbols), temperature-normalized (*p*CO_2bio/hydro_ – blue symbols) and *p*CO_2_-normalized (*p*CO_2thermal_ – red symbols) data collected in Santa Monica Bay at a depth of ~ 60 m.


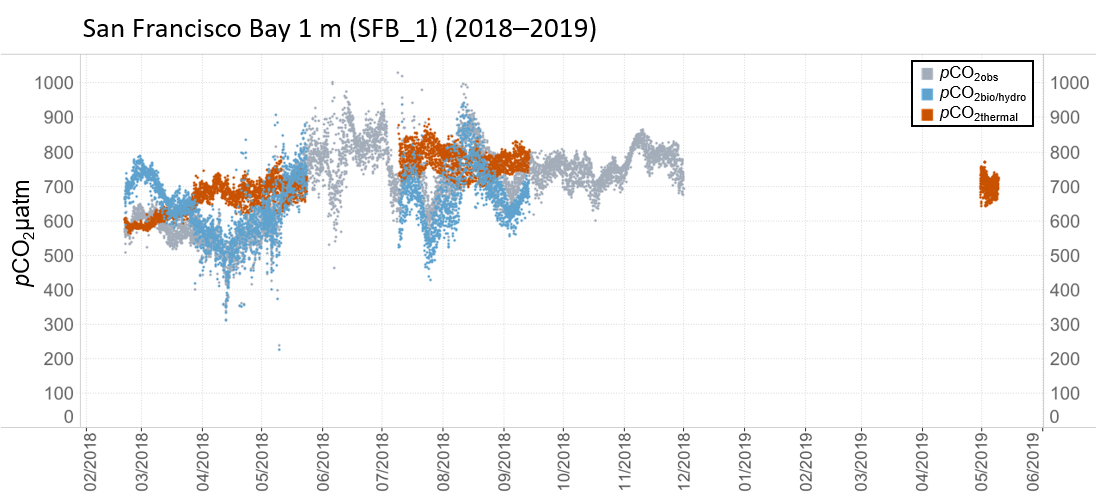


**Supplementary Figure 21.** Observed *p*CO_2_ (*p*CO_2obs_ – gray symbols), temperature-normalized (*p*CO_2bio/hydro_ – blue symbols) and *p*CO_2_-normalized (*p*CO_2thermal_ – red symbols) data collected in San Francisco Bay at a depth of ~ 1 m.


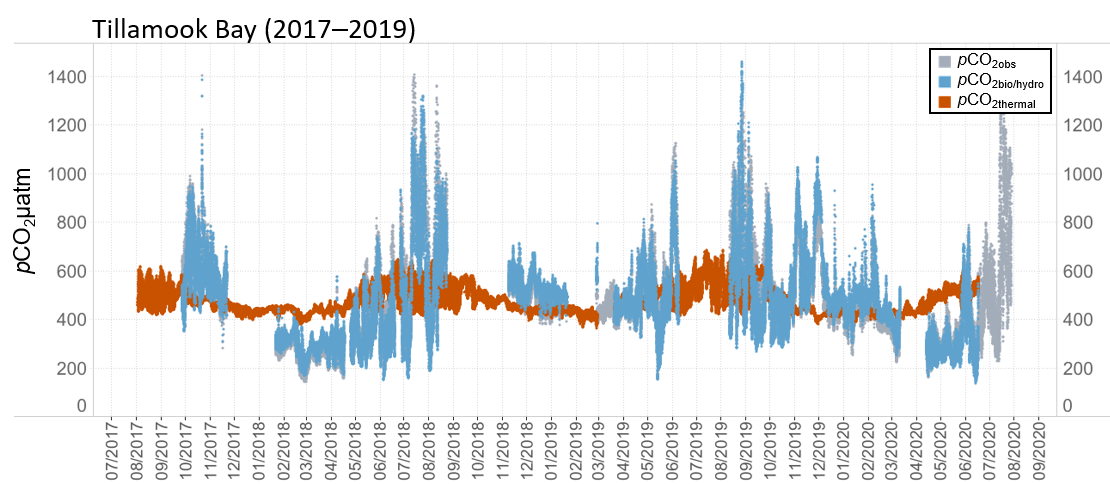


**Supplementary Figure 22.** Observed *p*CO_2_ (*p*CO_2obs_ – gray symbols), temperature-normalized (*p*CO_2bio/hydro_ – blue symbols) and *p*CO_2_-normalized (*p*CO_2thermal_ – red symbols) data collected in Tillamook Bay.


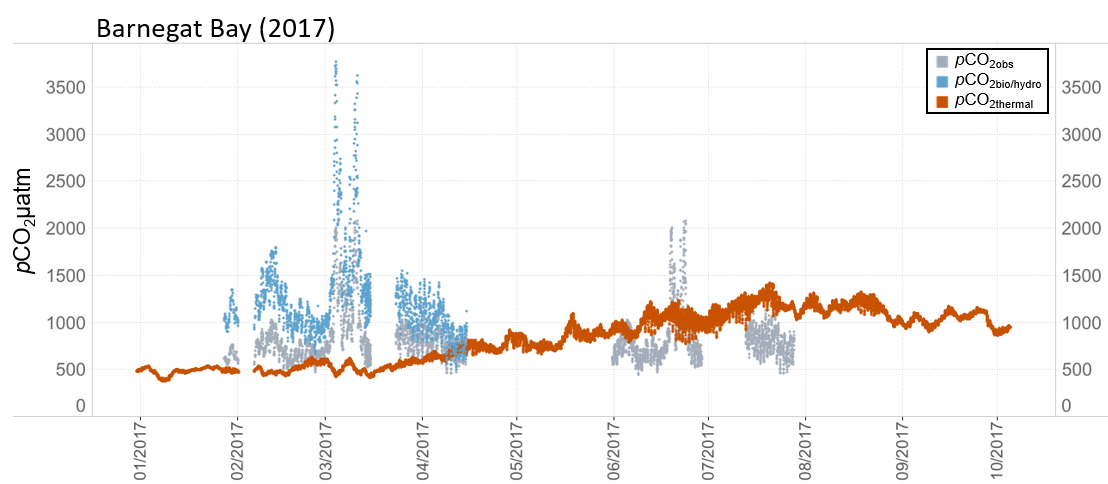


**Supplementary Figure 23.** Observed *p*CO_2_ (*p*CO_2obs_ – gray symbols), temperature-normalized (*p*CO_2bio/hydro_ – blue symbols) and *p*CO_2_-normalized (*p*CO_2thermal_ – red symbols) data collected in Barnegat Bay.


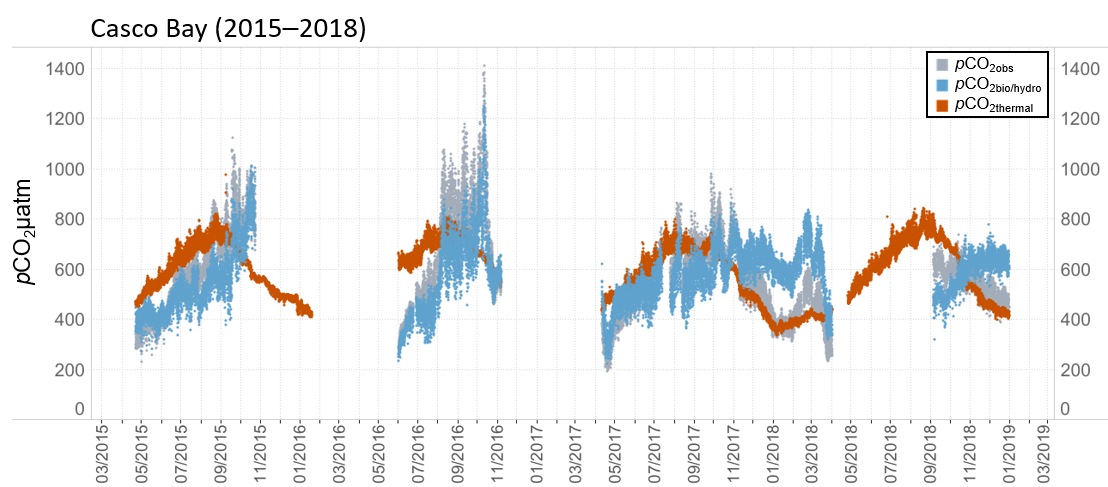


**Supplementary Figure 24.** Observed *p*CO_2_ (*p*CO_2obs_ – gray symbols), temperature-normalized (*p*CO_2bio/hydro_ – blue symbols) and *p*CO_2_-normalized (*p*CO_2thermal_ – red symbols) data collected in Casco Bay.


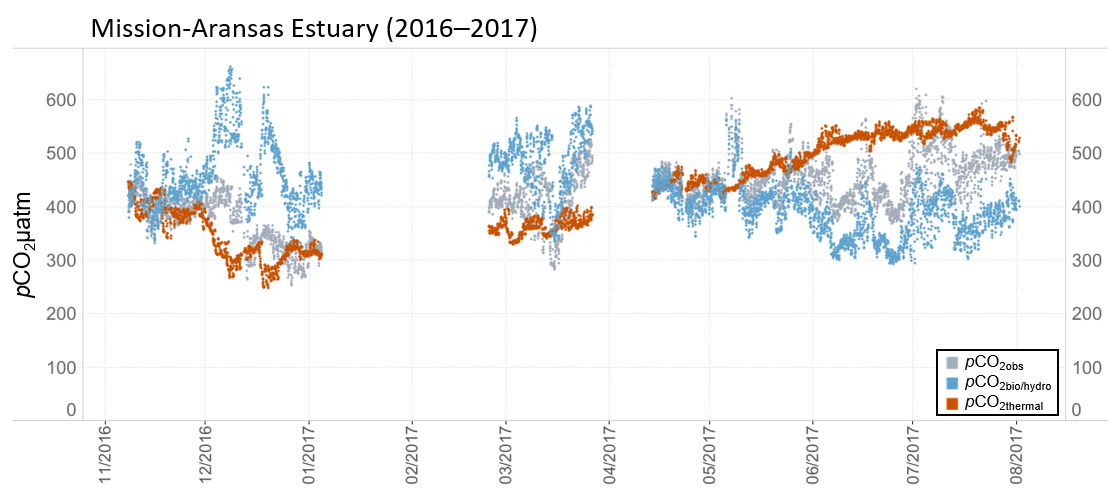


**Supplementary Figure 25.** Observed *p*CO_2_ (*p*CO_2obs_ – gray symbols), temperature-normalized (*p*CO_2bio/hydro_ – blue symbols) and *p*CO_2_-normalized (*p*CO_2thermal_ – red symbols) data collected in Mission-Aransas Estuary.


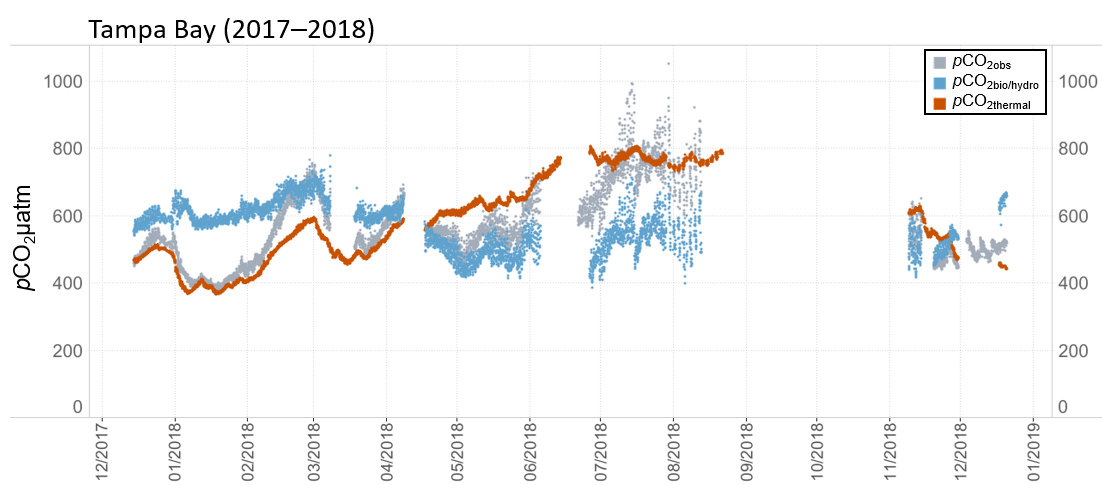


**Supplementary Figure 26.** Observed *p*CO_2_ (*p*CO_2obs_ – gray symbols), temperature-normalized (*p*CO_2bio/hydro_ – blue symbols) and *p*CO_2_-normalized (*p*CO_2thermal_ – red symbols) data collected in Tampa Bay.

# Supplementary Tables

**Supplementary Table 1.** Water body characteristics of seven of the U.S. Environmental Protection Agency’s (EPA) National Estuary Program sites.

| **Waterbody** | **Estuarine Drainage Area (km^2^)^a^** | **Watershed Population** | **Estuary Area (km^2^)^c^** | **Avg. Depth (m)** | **Tidal Height (m)** | **Land Use** | **Mean Monthly River Flow (m^3^ d^-1^)^c^** | **Mean Annual Air T (°C)** |
| --- | --- | --- | --- | --- | --- | --- | --- | --- |
| Santa Monica Bay | 1446^c^ | ~4,000,000^d^ | 2,767 | 95^e^ | 0.84^c^ | Urban: 75%^f^ Agr: 1%^f^ Forest: <1^f^ Wetland: 0%^f^  Other:23%^f^ | 173,878 | 16.1^c^ |
| San Francisco Bay | 5346^c^ | ~7,000,000^g^ | 1,309 | 6.4^c^ | 1.2^c^ | Urban: 6%^f^ Agr: 21%^f^  Forest: 53% ^f^  Wetland: 1%^f^  Other: 22%^f^ | 691,573 | 13.8^c^ |
| Tillamook Bay | 1428^c^ | ~7,500^h^ | 6 | 1.4^c^ | 1.2^c^ | Urban: 1%^f^ Agr: 5%^f^  Forest: 93%^f^ Wetland: <1%^f^ | 5,272,959 | 10.4^c^ |
| Barnegat Bay | 1581^c^ | ~560,000^i^ | 354 | 1.9^c^ | 0.15^c^ | Urban: 30%^j^ Agr: 1% ^j^ Forest: 40% ^j^ Wetland: 25% ^j^ | 1,321,322 | 11.6^c^ |
| Casco Bay | 2987.7^c^ | ~250,000^k^ | 84 | 12.0^f^ | ~3.0^c^ | Urban: 34%^k^ Agr: 8%^k^ Forest: 65%^k^ Wetland: 11%^k^  Other: 6%^k^ | 3,211,082 | 7.6^c^ |
| Mission-Aransas Estuary | 4,821^a^ | ~64,000^l^ | 527^n^ | 2^m^ | 0.15^m^ | Urban: 5%^l^ Agr: 34%^l^  Forest: 11%^l^  Wetland: 9%^l^ Other: 51%^l^ | 586,665 | 23.5^m^ |
| Tampa Bay | 5,698^b^ | ~3,000,000^b^ | 526 | 3.6^c^ | 0.37^c^ | Urban: 43%^b^ Agr: 20%^b^ Wetland: 7%  Other: 48%^b^ | 3,514,898 | 22.6^c^ |
| ^a^As defined in the NOAA Office of Ocean Resources Conservation and Assessment’s Coastal Assessment Framework (http://spo.nos.noaa.gov/projects/caf/caf.html) an Estuary Drainage Area is that component of an estuary’s entire watershed that empties directly into waters affected by the tides. | | | | | | | | |
| ^b^Latimer et al. (2018) | | | | | | | | |
| ^c^Engle et al. (2007) | | | | | | | | |
| ^d^Santa Monica Bay National Estuary Program | | | | | | | | |
| ^e^NOAA (1998) | | | | | | | | |
| ^f^National Estuarine Eutrophication Assessment: https://ian.umces.edu/neea/siteinformation.php | | | | | | | | |
| ^g^https://www.epa.gov/sfbay-delta/about-watershed | | | | | | | | |
| ^h^Lee and Brown (2009) | | | | | | | | |
| ^i^Barnegat Bay Partnership. 2012, Economic Value of the Barnegat Bay Watershed: https://www.barnegatbaypartnership.org/wp-content/uploads/wpallimport/files/BarnegatBayEconomic-report-112112.pdf | | | | | | | | |
| ^j^Barnegat Bay Partnership. 2016, State of the Bay Report: barnegatbaypartnership.org/wp-content/uploads/2017/08/BBP_State-of-the-Bay-book-2016_forWeb-1.pdf | | | | | | | | |
| ^k^Casco Bay Estuary Partnership. 2015, State of the Bay Report: https://www.cascobayestuary.org/wp-content/uploads/2016/03/State-of-the-Bay-Report-2015.pdf | | | | | | | | |
| ^l^Guneralp et al. (2013) | | | | | | | | |
| ^m^National Estuarine Research Reserves: http://cdmo.baruch.sc.edu/dges/ | | | | | | | | |
| ^n^Yao and Hu (2017) | | | | | | | | |

**Supplementary Table 2.** Deployment details for coastal acidification monitoring equipment in seven of the U.S. Environmental Protection Agency’s National Estuary Program sites.

| **Water Body** | **Data Collection Years** | **Water Depth (m)^a^** | **Sensor Location** | **Sensor Latitude (°N)** | **Sensor Longitude** | **Autonomous deployment details** | **Data collection interval** |
| --- | --- | --- | --- | --- | --- | --- | --- |
| Santa Monica Bay | 2016**–**17 | ~23 | ~15 m below surface | 33.7679 | -118.4331 | Instruments attached to custom built frame and suspended via thermistor string mooring located offshore of Palos Verdes Point. Location was chosen to characterize the ocean acidification and hypoxia in shallower water within the surface mixed layer, and within a few hundred meters of established kelp beds. The depth and location of the sensors were expected to minimize effects on measurements due to point discharges to Santa Monica Bay. | 60 mins |
|  | 2018**–**19 | ~70 | ~60 m below surface | 33.7129 | -118.3666 |  |  |
| San Francisco Bay | 2018**–**19 | 20–25 | ~1 m below surface | 37.8928 | -122.4469 | Surface buoy with intake 1 m below the surface (mbs) and deep water mooring (17 mbs) are located in Central San Francisco Estuary, in a deep former river channel that runs close to shore on the eastern side of the Tiburon peninsula at the interface between Central Bay (outer embayment) and San Pablo Bay (North Bay). | 15 mins |
|  | 2018**–**19 | 25–30 | ~17 m below surface | 37.8915 | -122.4467 |  | 60 mins |
| Tillamook Bay | 2017**–**19 | 2.4–5.2 | ~1 m above seafloor | 45.5538 | -123.9159 | Instruments mounted under dock at the Port of Garibaldi near the mouth of the estuary and wastewater treatment outfall. | 15 mins |
| Barnegat Bay | 2016**–**18 | 1.9–3.7 | ~0.5 m above seafloor | 39.5672 | -74.2449 | Instruments attached to piling at Morrison’s Marina in Beach Haven, New Jersey. | 15 mins |
| Casco Bay | 2015**–**20 | 1–5 | ~0.5 m above seafloor | 43.6512 | -70.2283 | Instruments housed in cage and deployed under pier in Portland channel near outlets of Fore and Presumpscot rivers; relatively urban part of estuary. | 60 mins |
| Mission-Aransas Estuary | 2016**–**17 | 6 | ~1 m below surface | 27.8381 | -97.0503 | Instruments housed in an external housing; water pumped from 1 m below surface; lower estuary (ship channel). | 60 mins |
| Tampa Bay | 2017**–**19 | 20–25 | ~2.5 m below surface | 27.6617 | -82.5947 | Instruments deployed on existing University of South Florida, Physical Oceanographic Real Time System (PORTS) station near lower, middle Tampa Bay. Location was selected to capture estuarine chemical signature during outgoing tides and Gulf of Mexico chemical signature during incoming tides. | 60 mins |
| ^a^Water depth at sensor location. Variability due to tidal range. | | | | | | | |

**Supplementary Table 3.** Seasonal averages and range of values for partial pressure of carbon dioxide (*p*CO_2_), pH, temperature, salinity, and dissolved oxygen (DO) in the seven water bodies. Data in cells expressed as the following: Minimum–Maximum (Mean | *1 SD*). GOM = Gulf of Mexico.

| **Coast** | | **Water Body (depth)^a^** |  | **Season^d,e^** | ***p*CO_2_ (μatm)** | **pH** | **T (°C)** | **Salinity** | **DO (mg/l)** |
| --- | --- | --- | --- | --- | --- | --- | --- | --- | --- |
| Pacific | | Santa Monica Bay (15 m) |  | Winter | 338 – 752 (590 \| *95*) | 7.80 – 8.10 (8.01 \|*0.05*) | 10.7–16.3 (14.2 \| *0.8*) | 32.6 – 33.5 (33.2 \| *0.8*) | 3.3 – 8.1 (5.3 \| *0.5*) |
|  |  | Santa Monica Bay (60 m) |  |  | 382–813 (590 \| *95*) | 7.73–7.98 (7.86 \| *0.05*) | 10.2–20.9 (12.9 \| *1.4*) | 33.3–33.7 (33.5 \| *0.1*) | 4.7–8.2 (7.9 \| *0.04*) |
|  |  | San Francisco Bay (1 m) |  |  | 507–777 (609 \| *50*) | ND | 10.2–12.0 (11.1 \| *0.3*) | 22.2–30.8 (26.5 \|*2.1*) | 7.3–8.9 (8.1 \| *0.3*) |
|  |  | San Francisco Bay (17 m) |  |  | ND | 7.78–7.92 (7.86 \| *0.03*) | 10.7–11.5 (11.1 \| *0.2*) | 23.3–31.2 (29.0 \| 1.9) | 7.3–8.5 (7.8 \| *0.3*) |
|  |  | Tillamook Bay |  |  | 143–716 (407\| *114*) | 7.61–8.31 (8.01 \|*0.06*) | 4.3–11.4 (9.9 \| *0.9*) | 0.03–32.8 (26.3 \| *5.5*) | 8.2–12.6 (9.5 \| *0.4*) |
| Atlantic | | Barnegat Bay |  |  | 438–1027 (700 \| *110*) | 7.90–8.27 (8.07 \| *0.07*) | -1.9–10.1 (4.4 \| *2.3*) | 27.4–30.7 (28.9 \| *0.7*) | 9.4–12.8 (10.9 \| *0.6*) |
|  |  | Casco Bay |  |  | 313–637 (356 \| *68*) | 7.96–8.08 (8.01 \| *0.02*) | -1.4–8.3 (4.3 \| *2.3*) | 11.3–31.6 (28.3 \| *3.1*) | 9.5–13.2 (11.6 \| *1.1*) |
| GOM | | Mission-Aransas Estuary |  |  | 251–457 (356 \| *48*) | 8.02 – 8.44 (8.22 \| *0.1*) | 11.5–22.5 (17.2 \| *2.2*) | 20.2–33.9 (27.4 \| *3.2*) | ND |
|  |  | Tampa Bay |  |  | 374–765 (495 \| *85*) | 7.82–8.29 (8.00 \| *0.1*) | 13.1–24.4 (17.8 \| *3.2*) | 28.0–33.1 (30.7 \| *0.9*) | 5.0–8.8 (7.5 \| *0.8*) |
| Pacific | | Santa Monica Bay (15 m) |  | Spring | 306–1417 (748 \| *206*) | 7.64–8.20 (7.89 \| *0.1*) | 10.0–16.9 (12.5 \| *1.2*) | 33.2–33.6 (33.4 \| *0.1*) | 2.6–7.2 (4.3 \| *1.0*) |
|  |  | Santa Monica Bay (60 m) |  |  | 575–1021 (874 \| *67*) | 7.71–7.93 (7.78 \| *0.03*) | 9.3–12.1 (10.2 \| *0.4*) | 33.4–34.0 (33.7 \| *0.1*) | 3.3–6.8 (4.2 \| *0.5*) |
|  |  | San Francisco Bay (1 m) |  |  | 238–925 (602 \| *89*) | 7.88–8.18 (8.03 \| *0.09*) | 10.5–18.6 (14.9 \| *1.5*) | 8.7–30.7 (20.9 \| *5.0*) | 6.1–10.0 (8.4 \| *0.4*) |
|  |  | San Francisco Bay (17 m) |  |  | ND | 7.45–8.13 (7.89 \| *0.09*) | 10.6–16.1 (13.1 \| *1.2*) | 10.2–31.6 (27.2 \| *3.2*) | 5.3–13.4 (7.9 \| *0.7*) |
|  |  | Tillamook Bay |  |  | 143–912 (400 \| *123*) | 7.53–8.30 (8.00 \| *0.11*) | 6.0–17.3 (11.4 \| *1.7*) | 0.03–33.2 (27.3 \| *4.2*) | 6.2–12.0 (9.1 \| *0.6*) |
| Atlantic | | Barnegat Bay |  |  | 454–2074 (869 \| *281*) | 7.68–8.25 (7.96 \| *0.1*) | 2.3–13.7 (7.6 \| *2.5*) | 21.4–31.2 (28.7 \| *1.0*) | 6.5–12.4 (9.2 \| *1.2*) |
|  | | Casco Bay |  |  | 191–576 (379 \| *71*) | 7.98–8.32 (8.12 \| *0.05*) | 0–23.2  (11.7 \| *5.2*) | 13.5–31.3 (27.8 \| *2.2*) | 9.5–13.3 (12.4 \| *0.5*) |
| GOM | | Mission-Aransas Estuary |  |  | 282–602 (436 \| *45*) | 7.82–8.31 (8.09 \| *0.09*) | 18.2–28.1 (23.7 \| *2.8*) | 20.3–35.8 (30.2 \| *2.6*) | ND |
|  |  | Tampa Bay |  |  | 456–756 (558 \| *56*) | 7.72–8.07 (7.94 \| *0.04*) | 18.1–27.4 (23.5 \| 2.7) | 28.6–34.0 (32.0 \| *0.9*) | 3.9–7.8 (6.6 \| *0.5*) |
| Pacific | | Santa Monica Bay (15 m) |  | Summer | 335–1383 (554 \| *140*) | 7.73–8.15 (7.97 \| *0.1*) | 11.1–22.5 (14.6 \| *1.8*) | 33.1–33.5 (33.3 \| *1.8*) | 3.2–6.7 (5.3 \| *0.6*) |
|  |  | Santa Monica Bay (60 m) |  |  | 574–1021 (874 \| *67*) | 7.71–7.93 (7.78 \| *0.3*) | 9.4–12.1 (10.2 \| *0.4*) | 33.4–34.0 (33.7 \| *1.0*) | 3.3–6.8 (4.2 \| *0.5*) |
|  |  | San Francisco Bay (1 m) |  |  | 461–1028 (768 \| *83*) | 7.68–7.84 (7.73 \| *0.2*) | 12.7–21.8 (17.6 \| *1.4*) | 10.6–31.9 (25.4 \| *4.7*) | 4.7–10.2 (7.3 \| *0.7*) |
|  |  | San Francisco Bay (17 m) |  |  | ND | 7.02–8.00 (7.67 \| *0.1*) | 12.2–20.4 (16.3 \| *1.5*) | 14.1–32.1 (28.9 \| *2.4*) | 4.9–14.9 (7.0 \| *0.6*) |
|  |  | Tillamook Bay |  |  | 171–1983 (691 \| *304*) | 7.36–8.29 (7.85 \| *0.2*) | 8.5–20.5 (13.3 \| *2.4*) | 0.05–33.8 (31.3 \| *1.9*) | 3.3–11.9 (7.7 \| *0.2*) |
| Atlantic | | Barnegat Bay |  |  | 438–2074 (809 \| *249*) | 7.60–8.16 (7.87 \| *0.1*) | 14.8–29.3 (23.5 \| *2.5*) | 25.4–31.7 (29.0 \| *1.4*) | 4.1–9.6 (6.7 \| *0.9*) |
|  | | Casco Bay |  |  | 264–1076 (590 \| *141*) | 7.35–8.20 (7.86 \| *0.2*) | 9.5–20.4 (15.1 \| *2.2*) | 22.5–31.4 (29.7 \| *2.15*) | 5.6–13.4 (10.5 \| *1.0*) |
| GOM | | Mission-Aransas Estuary |  |  | 368–620 (461 \| *51*) | 7.79–8.18 (8.05 \| *0.06*) | 27.2–31.7 (29.7 \| *0.8*) | 21.4–37.7 (32.5 \| *0.75*) | ND |
|  | | Tampa Bay |  |  | 538–1050 (715 \| *80*) | 7.54–8.09 (7.85 \| *0.06*) | 27.1–31.8 (30.3 \| *0.9*) | 18.8–32.6 (28.9 \| *2.5*) | 1.3–7.8 (5.7 \| *0.6*) |
| Pacific | | Santa Monica Bay (15 m) |  | Fall | 342–602 (541 \| *58*) | 7.89–8.12 (8.02 \| *0.04*) | 12.3–22.8 (15.5 \| *1.6*) | 33.1–33.4 (33.3 \| *0.05*) | 4.3–6.0 (5.4 \| *0.3*) |
|  |  | Santa Monica Bay (60 m) |  |  | 438–678 (553 \| *55*) | 7.29–7.98 (7.74 \| *0.2*) | 11.4–16.8 (13.0 \| *0.7*) | 33.2–33.6 (33.5 \| *0.04*) | 5.6–7.9 (6.8 \| *0.5*) |
|  |  | San Francisco Bay (1 m) |  |  | 600–864 (751 \| *45*) | 7.43–7.83 (7.75 \| *0.12*) | 14.1–22.2 (17.8 \| *1.5*) | 20.1–31.8 (27.2 \| *2.4*) | 4.0–10.2 (7.1 \| *0.8*) |
|  |  | San Francisco Bay (17 m) |  |  | ND | 7.29–7.90 (7.68 \| *0.03*) | 14.4–20.4 (17.1 \| *1.3*) | 23.4–31.7 (29.2 \| *1.9*) | 5.4–8.0 (6.6 \| *0.3*) |
|  |  | Tillamook Bay |  |  | 260–2586 (612 \| *217*) | 7.21–8.27 (7.96 \| *0.1*) | 8.9–18.6 (11.7 \| *1.2*) | 0.3–33.7 (29.3 \| *4.2*) | 3.50–11.2 (8.4 \| *1.0*) |
| Atlantic | | Barnegat Bay |  |  | ND | 7.65–8.20 (7.89 \| *0.1*) | 17.5–24.8 (21.5 \| *1.8*) | 27.2–30.8 (28.7 \| *0.6*) | 5.3–9.3 (7.1 \| *0.8*) |
|  |  | Casco Bay |  |  | 420–1409 (697 \| *145*) | 7.16–8.04 (7.74 \| *0.2*) | 4.9–23.9 (13.1 \| *3.19*) | 17.9–32.1 (29.8 \| *1.6*) | 5.6–12.7 (9.5 \| *1.1*) |
| GOM | | Mission-Aransas Estuary |  |  | 331–489 (400 \| *25*) | 8.03–8.38 (8.18 \| *0.05*) | 18.9–25.3 (22.5 \| *1.2*) | 18.3–35.3 (27.6 \| *3.7*) | ND |
|  |  | Tampa Bay |  |  | 438–650 (500 \| *50*) | 7.76–8.02 (7.91 \| *0.06*) | 18.8–25.9 (22.9 \| *1.9*) | 25.4–32.5 (29.7 \| *1.2*) | 4.2–5.5 (4.7 \| *0.2*) |
|  | ^a^Depth noted for systems with multiple deployments. | | | | | | | | |
|  | ^b^Sensors removed from water between January 20, 2016 and June 2, 2016. | | | | | | | | |
|  | ^c^Deployment halted due to lack of infrastructure after hurricane. | | | | | | | | |
|  | ^d^For water bodies where more than one year of data are available, seasonal values were calculated by averaging data from multiple years. | | | | | | | | |
|  | ^e^Northern hemisphere meteorological seasons: Winter – DJF; Spring – MAM; Summer – JJA; Fall – SON | | | | | | | | |
|  | ND: No Data | | | | | | | | |

**REFERENCES**

CASCO Bay Estuary Partnership (CBEP) (2015). State of the Bay 2015 Report. Portland, ME: Casco Bay Estuary Partnership (CBEP).

Dickson, A. G., Sabine, C. L., and Christian, J.R., eds (2007). Guide to best practices for ocean acidification CO2 measurements: North Pacific Marine Science Organization, Special Publication 3, 191 p. Available online at: https: //www.nodc.noaa.gov/ocads/oceans/Handbook_2007/Guide_all_in_one.pdf. (accessed April 10, 2018).

Engle, V. D., Kurtz, J. C., Smith, L. M., Chancy, C., and Bourgeois, P. (2007). A classification of U.S. estuaries based on physical and hydrologic attributes. Environ. Monit. Assess. 129, 397–412. doi: 10.1007/s10661-006-9372-9

Guneralp, B., Guneralp, I., Castillo, C. R., and Filippi, A. M. (2013). Land change in the mission-aransas coastal region, texas: implications for coastal vulnerability and protected areas. Sustainability 5, 4247–4267. doi: 10.3390/su5104247

Latimer, J. S., Trettin, C. C., Bosch, D. D., and Lane, eds (2019). Working Watersheds And Coastal Systems: Research And Management For A Changing Future—Proceedings Of The Sixth Interagency Conference On Research In The Watersheds. July 23-26, 2018, Shepherdstown, WV. e-Gen. Tech. Rep. SRS-

243. Asheville, NC: U.S. Department of Agriculture Forest Service, Southern Research Station., 211.

Lee, H. II, and Brown, C.A. (eds.) (2009). Classification of Regional Patterns of Environmental Drivers A\] and Benthic Habitats in Pacific Northwest Estuaries. EPA/600/R-09/140. Washington, DC: U.S. EPA, Office of Research and Development.

National Oceanic and Atmospheric Administration (NOAA) (1998). NOAA’s Estuarine Eutrophication Survey, Volume 5: Pacific Coast Region. Silver Spring, MD: Office of Ocean Resources Conservation and Assessment, 75.
